# Mitochondrial Calcium Uniporter Drives Chemoresistance in Pancreatic Cancer via Glutathione‐Mediated Stemness Maintenance

**DOI:** 10.1002/advs.202507346

**Published:** 2026-01-21

**Authors:** Zekun Li, Chenyang Meng, Guangcong Shen, Yueying Shan, Junjin Wang, Diliyaer Abudukeremu, Rui Zhao, Bo Ni, Chao Xu, Xiaofan Guo, Jun Yu, Kaiyuan Wang, Shengyu Yang, YunZhan Li, Yongjie Xie, Tianxing Zhou, Jihui Hao, Xiuchao Wang

**Affiliations:** ^1^ Pancreas Center National Clinical Research Center for Cancer State Key Laboratory of Druggability Evaluation and Systematic Translational Medicine Tianjin Medical University Cancer Institute and Hospital Tianjin Key Laboratory of Digestive Cancer Tianjin's Clinical Research Center for Cancer Tianjin China; ^2^ Gastrointestinal Cancer Institute and Pancreatic Disease Institute The Affiliated Hospital of Qingdao University Qingdao China; ^3^ Tianjin Medical University Tianjin China; ^4^ Department of Anesthesiology National Clinical Research Center for Cancer State Key Laboratory of Druggability Evaluation and Systematic Translational Medicine Tianjin Medical University Cancer Institute and Hospital Tianjin's Clinical Research Center for Cancer Tianjin China; ^5^ Department of Cellular and Molecular Physiology Penn State College of Medicine Hershey Pennsylvania USA; ^6^ Institute of Translational Medicine China Pharmaceutical University Nanjing China

**Keywords:** chemoresistance, glutathione synthesis, mitochondrial calcium uniporter, pancreatic ductal adenocarcinoma, stemness maintenance

## Abstract

Pancreatic ductal adenocarcinoma (PDAC) remains a lethal malignancy with poor prognosis due to chemoresistance. Using integrative single‐cell RNA sequencing, we identified that the upregulation of mitochondrial calcium uniporter (MCU) may contribute to chemoresistance and stemness maintenance in PDAC. MCU was highly expressed in chemotherapy‐resistant PDAC tumors and correlated with enhanced cancer stem cell properties. Mechanistically, MCU‐mediated mitochondrial Ca^2+^ influx triggered endoplasmic reticulum (ER) stress and the downstream PERK‐eIF2α pathway. This cascade activated ATF4 and NRF2, which enhanced the transcriptional regulation of PSAT1 and SLC7A11. These changes promoted *de novo* glutathione (GSH) synthesis to scavenge reactive oxygen species (ROS) and sustain stemness. Genetic knockdown or pharmacological inhibition of MCU disrupted GSH synthesis, suppressed stemness, and restored sensitivity to nab‐paclitaxel plus gemcitabine (AG). High‐throughput screening identified MCU inhibitor NB‐598, which synergized with AG to inhibit tumor growth in preclinical models. These findings offer a potential novel therapeutic strategy to address chemoresistance in PDAC.

## Graphical abstract

PDAC has a poor prognosis due to chemoresistance. We revealed that MCU upregulation is associated with chemoresistance and stemness in PDAC. MCU‐mediated Ca^2^^+^ influx induced ER stress, activating the PERK‐ATF4/NRF2 axis to enhance PSAT1/SLC711 expression and glutathione synthesis, reducing ROS and maintaining stemness. The newly identified MCU inhibitor NB‐598 suppressed MCU‐mediated stemness and restored sensitivity to AG chemotherapy.

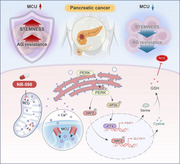

## 1 Introduction

Pancreatic ductal adenocarcinoma (PDAC) remains one of the most lethal malignancies, characterized by a dismal 5‐year survival rate, largely attributable to therapeutic resistance [1, 2]. While combination chemotherapy with albumin‐bound paclitaxel plus gemcitabine (AG) has emerged as the standard first‐line regimen, its clinical benefits remain limited due to intrinsic and acquired chemoresistance. This underscores an urgent need to unravel the molecular mechanisms that underlie chemoresistance in PDAC.

Cancer stemness describes the stem‐like phenotypic features of tumor cells, including self‐renewal capacity, multilineage differentiation potential, and enhanced drug efflux capabilities [3, 4]. Accumulating evidence confirms that the maintenance of cancer stemness is a key mediator of therapeutic resistance and tumor recurrence following treatment [5, 6]. Notably, recent studies have highlighted the interplay between tumor metabolic reprogramming and the maintenance of cancer stemness. Our previous research revealed that PDAC cells dynamically rewire iron metabolism and amino acid utilization pathways to support stemness‐associated traits, suggesting that targeting these metabolic vulnerabilities could be a viable approach to overcome chemoresistance [7, 8]. However, the upstream regulatory factors that coordinate these metabolic adaptations remain largely undefined.

Mitochondrial calcium uniporter (MCU), the pore‐forming core subunit of the MCU complex, plays a pivotal role in maintaining Ca^2+^ homeostasis between the cytosol and mitochondria. This homeostasis is essential for sustaining energy metabolism and other cellular processes, which are indispensable for preserving cancer stemness [9, 10]. MCU is frequently upregulated in diverse malignancies, such as breast cancer and colorectal cancer [11, 12], where it drives tumor progression by orchestrating mitochondrial reactive oxygen species signaling and reprogramming oxidative phosphorylation. Despite these insights, whether MCU regulates the self‐renewal capacity and chemoresistance of PDAC remains unexplored.

In the present study, we demonstrated that elevated MCU expression correlated with a diminished chemotherapeutic response in PDAC patients. Mechanistically, we show that MCU promotes *de novo* serine synthesis and glutathione (GSH) metabolism, which in turn enhance tumor stemness and chemoresistance. Furthermore, we validate the potential therapeutic value of targeting MCU in preclinical models, proposing MCU as a novel candidate for improving PDAC treatment outcomes.

## 2 Materials and Methods

### 2.1 Single‐Cell RNA Sequencing Analysis

Based on single‐cell transcriptome sequencing technology, this study systematically established an analytical pipeline from raw data processing to functional interpretation. Experimental data were first processed using the Scrublet algorithm (default parameters) to remove doublet interference. A dual quality control standard was applied: low‐quality cells with either > 6000 or < 200 expressed genes were excluded, along with samples exhibiting a mitochondrial gene unique molecular identifiers (UMI) ratio exceeding 8%, to eliminate the impact of low‐quality cells. Seurat integration was employed for systematic analysis. Data normalization was performed via the NormalizeData function to scale gene expression, followed by FindVariableFeatures for highly variable gene selection and independent analytical unit construction.

During the feature extraction phase, principal component analysis (PCA, 20 dimensions), UMAP nonlinear dimensionality reduction, and *t*‐SNE nonlinear dimensionality reduction were applied to the integrated dataset for dimensionality reduction and spatial feature reconstruction. After technical noise correction via variance scaling (ScaleData), cell clustering and identification were performed using k‐nearest neighbor graph construction (FindNeighbors) and the Louvain algorithm (FindClusters). Dimension selection combined ElbowPlot inflection point interpretation and Pearson's correlation hierarchical clustering. Clustering results were visualized using UMAP. Finally, subpopulation‐specific markers were screened via FindAllMarkers, with stringent thresholds for differential gene analysis (adjusted *p*‐value <0.05, log_2_ FC >0.5). The FindMarkers function was used to identify significantly differentially expressed genes (DEGs) between conditions and Gene Ontology (GO) and Gene Set Enrichment Analysis (GSEA) were performed to explore potential biological implications.

### 2.2 High‐Dimensional Weighted Gene Co‐Expression Network Analysis (hdWGCNA)

hdWGCNA was used to examine gene co‐expression patterns in high‐dimensional, sparse single‐cell transcriptome data. Cell subpopulations were first identified using the Seurat toolkit, and WGCNA objects were prepared via SetupForWGCNA. Metacells were generated using MetacellsByGroups to reduce data noise. During network construction, a soft thresholding strategy was applied: the TestSoftPowers function evaluated the effects of different soft thresholds, visualized via PlotSoftPowers, to select the optimal threshold. Module eigengenes were calculated to identify core genes within each module. The co‐expression network was ultimately constructed using the Construct Network function.

### 2.3 Pseudotime Analysis

Pseudotime analysis was performed to reveal dynamic patterns of cell state evolution. The Slingshot algorithm, based on minimum spanning tree principles, integrated transcriptomic features and spatial topology by inputting a UMI matrix (representing gene expression) and cell cluster labels. This method modeled pseudotemporal trajectories for clustered cell subpopulations, constructing probabilities of cell state transitions and quantifying gene expression continuity to simulate temporal changes and elucidate developmental directions.

### 2.4 AUCell Gene Set Scoring and Evaluation of Gene Importance

The AUCell package was used to assess the activity of target pathway gene sets across cells. By comparing the distribution of gene expression within each cell, the relative ranking of target genes in the overall expression profile was calculated. Cells with highly expressed target genes received elevated activity scores. After batch effect correction, spatial distribution maps or developmental trajectory projections were used to visualize the localization and dynamics of high‐activity cell populations, highlighting functional heterogeneity. A novel statistical method (scDist) was also applied to evaluate gene importance. The scDist algorithm estimated distances between conditional means of cell types, incorporated sample variability via a linear mixed model, and defined gene importance in specific cells based on principal component regression and gene loadings. For stemness evaluation, using the stemness‐associated gene signature as a reference (see Table S2 for details), the AUCell algorithm was applied to compute a stemness‐associated gene set activity score for each individual cell. Subsequently, epithelial cells were stratified into High‐stemness and Low‐stemness subpopulations using the median AUCell score across all epithelial cells in the cohort as the cutoff value.

### 2.5 Patient and Sample Collection

All patients in this study were recruited from the Department of Pancreatic Oncology, Tianjin Medical University Cancer Institute and Hospital. Written informed consent was obtained from all participants for the use of their biological specimens and clinical data in future research, in strict adherence to the ethical guidelines of the Ethics Committee of Tianjin Medical University Cancer Institute and Hospital (Approved No.: EK20150087, EK20220153) and the Declaration of Helsinki. Our retrospective study included three independent patient cohorts between 2016 and 2022. Cohort 1 included 68 treatment‐naive patients with pathologically confirmed metastatic PDAC diagnosed via percutaneous biopsy. Cohort 2 included 49 patients with radiologically defined borderline resectable or locally advanced PDAC diagnosed via percutaneous biopsy. All patients received chemotherapy with the AG regimen for 3 consecutive cycles. Tumor response was evaluated by contrast‐enhanced computed tomography (CE‐CT) per Response Evaluation Criteria in Solid Tumors (RECIST) version 1.1 within 1 week after completing 3 cycles of chemotherapy. Cohort 3 included 129 treatment‐naive patients who underwent radical R0 resection for PDAC. All patients underwent 6 cycles of postoperative AG chemotherapy with regular follow‐up. The final follow‐up date was January 1, 2025. In addition, 45 consecutive cases of fresh PDAC tissues were prospectively collected during surgery between January 2024 and November 2024. Detailed information on patient baseline characteristics and inclusion/exclusion criteria is provided in Tables S3–S6.

### 2.6 Primary Human Pancreatic Cancer Cells Culture and Transfection

Human pancreatic tumors were obtained during surgery with written informed consent from all the patients. Mycoplasma contamination was excluded in these cell lines. Cells were cultured at 37°C in a humidified atmosphere of 95% air and 5% CO2 with basic DMEM medium supplemented with 10% FBS. Low‐passage (< 10 passages) primary cancer cells were used for later experiments. Patient clinical data is provided in Table S7.

Human MCU cDNA (NM_138357.3) was cloned into a pLV expression vector (pLV‐MCU). The empty pLV vector was used as a control. Lentiviral transductions were performed according to standard procedures. For the stable knockdown cell lines, shRNA sequences were designed using the Sigma–Aldrich shRNA designer. Three recommended sequences targeting the gene of interest were synthesized and cloned into the pLV‐RNAi‐Puro vector (Biosettia). For the generation of stable knockout cell lines, sgRNA sequences were designed using the CRISPR Design Tool (crispr.mit.edu). Three highly specific sgRNA sequences were synthesized and cloned into the lentiCRISPR v2‐Puro vector (Addgene) following the Zhang lab protocol. Among the three shRNA sequences and three sgRNA sequences, the most effective one was selected for subsequent experiments. For details, refer to Table S8.

### 2.7 Construction of Subcutaneous Patient‐Derived Xenograft (PDX) Tumor Model

A subcutaneous PDX tumor model was constructed in mice as an experimental model that more accurately reflected the characteristics of primary tumors. Initially, fresh tumor tissue samples were obtained from cancer patients, representing the heterogeneity and complexity of primary cancers. The patient's tumor tissue was then implanted subcutaneously into immunodeficient mice. The growth rate and characteristics of the transplanted tumors were regularly monitored and measured. Once the tumors reached an appropriate size, a portion of the tumor tissue was collected for expansion of the PDX model, ensuring its representativeness and stability. The Animal Ethical and Welfare Committee (AEWC) of Tianjin Medical University Cancer Institute and Hospital approved all animal experiments (Approved No.: 2023086).

### 2.8 Spheroid Formation Assay

Cells were re‐suspended in serum‐free basal medium (2000 cells/mL), seeded into a low‐adhesion 6‐well plate, and cultured for 2–3 weeks. The number of spheroids with a diameter greater than 75 µm was counted under a microscope.

### 2.9 Flow Cytometry

Tumor cells were isolated from human and mouse xenografts and subjected to flow cytometric analysis. Briefly, tumor tissue samples were digested in 1 mg/mL collagenase I (Sigma‐Aldrich, C5138‐1G), 20 U/mL DNase I (Solarbio, D8071), and 0.1 mg/mL hyaluronidase 37°C for 1–2 h on a shaker at 80 rpm. Every 30 min, the tissue was aspirated and dispensed with a 1 mL pipette until no significant resistance was observed, indicating complete digestion. The cells were then filtered through a 70 µm cell filter, washed twice with HBSS (Solarbio, H1020), and resuspended in a single‐cell suspension. To analyze stem cell‐related surface markers, cells were incubated with the specified antibodies in the dark at room temperature for 30 min. For intracellular cytokine detection, cells were incubated with a Leukocyte Activation Cocktail (BD Biosciences) for 4 h, followed by staining with a Fixation/Permeabilization Kit (BD Biosciences). Flow cytometric data were acquired on a Beckman Coulter flow cytometer, and analysis was performed using FlowJo. The following antibodies were used for flow cytometry can be found in Table S9.

### 2.10 Western Blotting

Cells were lysed on ice for 5 min in a SDS‐NP40 buffer (50 mM Tris, pH 8.0, 150 mM NaCl, 1% NP‐40, 1% SDS, and 1 mM protease inhibitor cocktail). Lysates were collected by scraping the cells from the plate, followed by brief sonication (three cycles), then centrifuged at 15 000 × g for 10 min at 4°C. Protein samples (20–40 µg) were separated by SDS‐PAGE and transferred to a PVDF membrane. The membrane was blocked with 5% (w/v) non‐fat dry milk in Tris‐buffered saline with 0.05% Tween‐20 (TBS‐T) for 30 min at room temperature. After blocking, the membrane was incubated with primary antibodies at 4°C for 20 h, followed by a 1 h incubation with secondary antibodies at room temperature. Details of the primary antibodies used can be found in Table S10.

### 2.11 Statistical Methods

Statistical analyses were performed using SPSS Statistics version 26.0 and R software version 4.4.3. Data are presented as the mean value ± SD from 3 biological replicates. To evaluate differences between experimental groups, analysis of variance (ANOVA) followed by Tukey's multiple comparison test was used. The Student's *t‐*test was applied for comparing mean values between groups to determine statistical significance. For DEGs analyses, the false discovery rate (FDR) correction was employed to adjust *p*‐values, with a threshold set at FDR <0.05. Survival analysis was conducted using the Kaplan–Meier method, and survival curves were compared using the log‐rank test.

### 2.12 Data Availability 

Deidentified single‐cell RNA sequencing raw data are available from the National Omics Data Encyclopedia (NODE) under accession numbers OEP00006728 (https://www.biosino.org/node/project/detail/OEP00006728) and OEP00006742 (https://www.biosino.org/node/project/detail/OEP00006742). Further information related to the data reported in this paper can be acquired from the lead contact Xiuchao Wang (wangxiuchao2008@163.com) upon reasonable request.

## 3 Results

### 3.1 Single‐Cell RNA Sequencing Analysis Identifies MCU as a Candidate Regulator Driving Stemness Maintenance and Chemoresistance in PDAC

**FIGURE 1:**
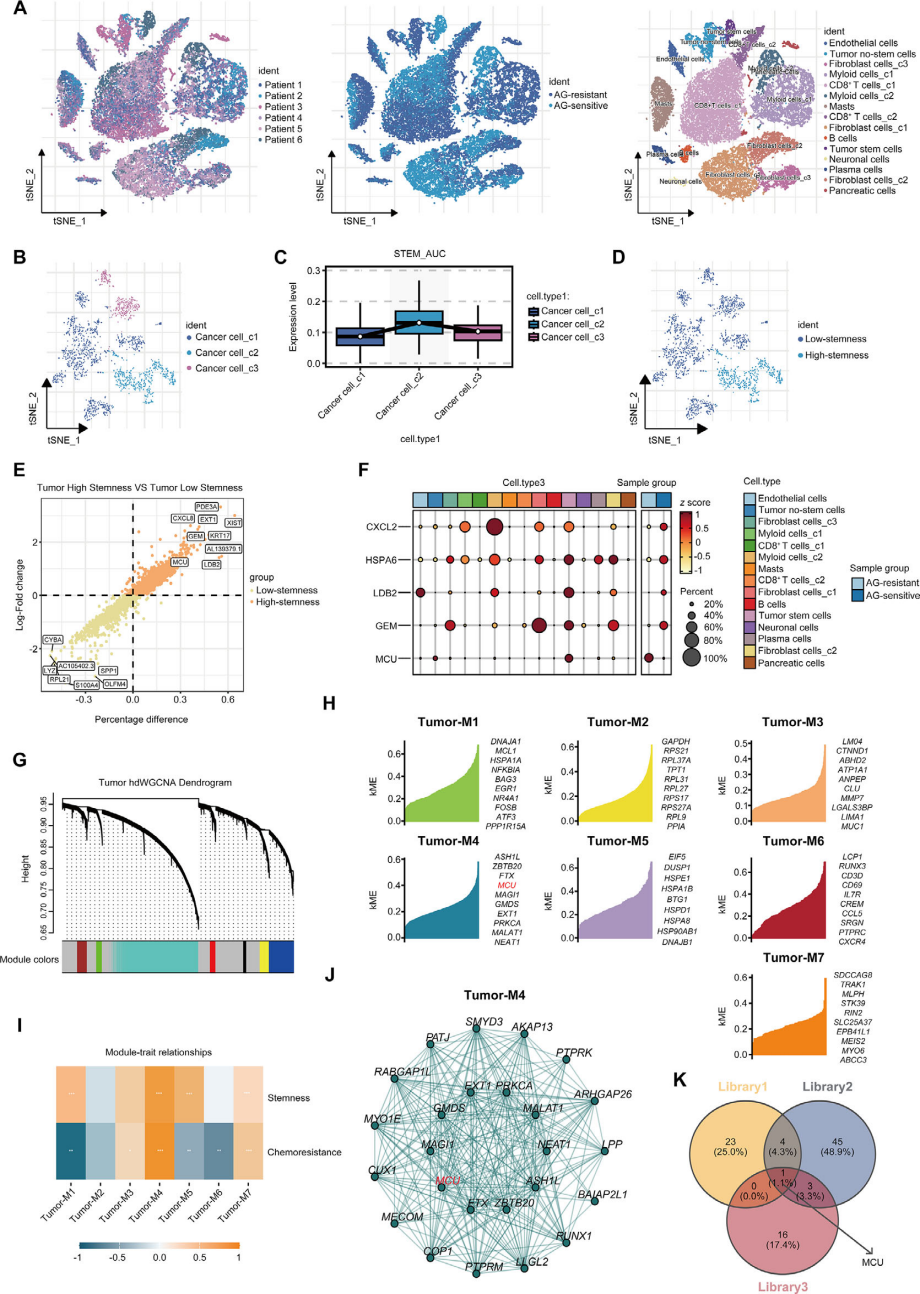
MCU is the module eigengene characterized by high stemness and chemoresistance. (A) t‐SNE plot showing cell clustering analysis of specimens from six patients grouped by the neoadjuvant therapy response. Annotation visualization of all samples was conducted using Seurat's t‐SNE algorithm. (B) t‐SNE plot showing the ductal cell populations after re‐clustering and sub‐clustering. (C) Boxplot showing the expression levels of stemness‐associated genes in three clusters. (D) t‐SNE plot showing the ductal cell clusters grouped by high‐stemness and low‐stemness. (E) Scatter plot showing differentially expressed genes (DEGs) between the high‐stemness and low‐stemness groups. (F) Bubble plot showing the expression levels of the indicated genes across different cell subpopulations and ductal cell clusters grouped by the neoadjuvant therapy response of patients. (G) hdWGCNA dendrogram of Cluster 2 showing seven unsupervised clustered co‐expression modules. (H) Module eigengene (ME) distribution patterns in seven co‐expression modules based on kME values. (I) Heatmap showing the expression patterns of stemness‐associated and chemoresistance genes across seven co‐expression modules. (J) Gene co‐expression network showing hub genes in the Tumor‐M4 module prioritized by intramodular connectivity. (K) Venn diagram showing MCU as the hub gene intersecting Library 1, 2, and 3. Data in (C,E,I) were analyzed using one‐way ANOVA and two‐sample, two‐tailed unpaired Student's *t*‐test. ns not significant, ^*^ *p* <0.05, ^**^ *p* <0.01, ^***^ *p* <0.001 and ^****^ *p* <0.0001.

Primary chemoresistance severely impairs the efficacy of neoadjuvant therapy for PDAC. To dissect the underlying molecular mechanisms, we designed and implemented a multi‐step screening strategy. First, single‐cell RNA sequencing was performed on tumor specimens from six PDAC patients, three with sensitivity to the AG chemotherapy regimen and three with resistance. After quality control and UMAP clustering, three distinct epithelial cell clusters were identified (Figure 1A,B). Comparative analysis of these clusters revealed 28 genes that were significantly upregulated in AG‐resistant populations; this gene set was designated as Library 1 (chemoresistance‐associated gene set). Subsequently, we integrated and filtered a subset of core stemness‐associated genes from the Kyoto Encyclopedia of Genes and Genomes (KEGG) database and defined them as a stemness‐associated gene signature. Using this signature as a reference, we classified the epithelial cell clusters by AUCell score, Cluster 2 was defined as the high‐stemness subpopulation, while Clusters 1 and 3 were categorized as low‐stemness subpopulations (Figure 1C,D). Further differential expression analysis identified 52 genes upregulated in the high‐stemness cluster, which formed Library 2 (stemness‐associated gene set) (Figure 1E,F). To explore the co‐expression relationships of genes in the high stemness subpopulation, we performed high‐dimensional weighted gene co‐expression network analysis (hdWGCNA) on cells from Cluster 2. This analysis resolved seven independent co‐expression modules, among which the Tumor‐M4 module showed the strongest correlation with both stemness‐associated and chemoresistance‐associated genes (Figure 1G–I). Intramodular connectivity analysis further identified the module eigengene (first principal component) of the Tumor‐M4 module as Library 3 (Figure 1J). Intersecting Library 1, 2, and 3 pinpointed mitochondrial calcium uniporter (MCU) as the sole overlapping hub gene (Figure 1K).

Validation using an independent single‐cell dataset (GSA: CRA001160) confirmed these findings [13] (Figure S1A). After dimensionality reduction and clustering, epithelial cells were stratified into high‐stemness and low‐stemness subpopulations (Figure S1B,C). Consistently, MCU expression was significantly elevated in high‐stemness clusters (Figure S1D,E). Collectively, these results establish MCU as a candidate regulator of stemness maintenance and AG regimen resistance in PDAC.

### 3.2 MCU in PDAC Progression and Chemoresistance

**FIGURE 2:**
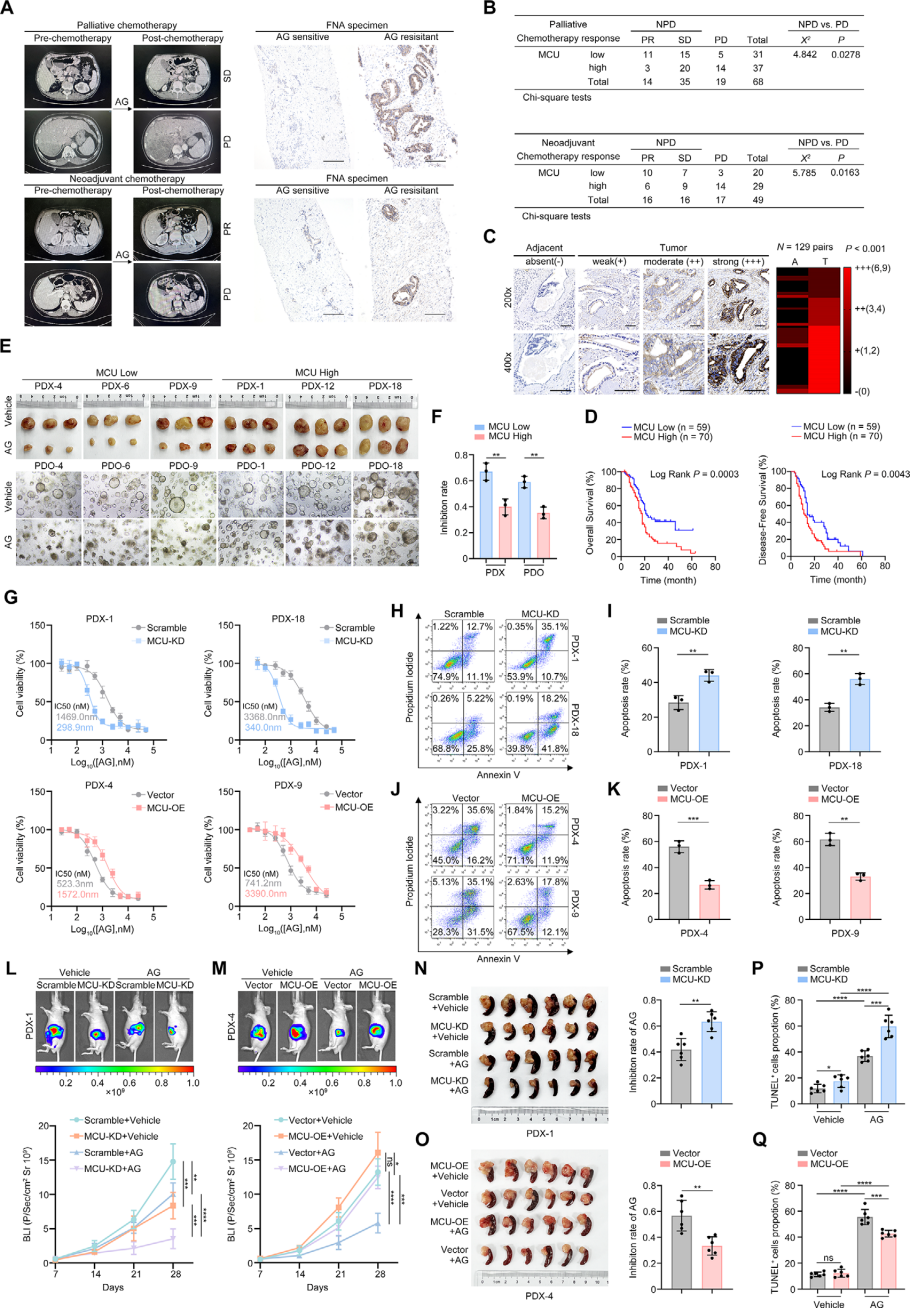
Both constitutive and acquired MCU expression drive resistance to AG therapy in PDAC. (A) Representative CT scans and IHC staining images from PDAC patients treated with AG palliative chemotherapy (*n* = 68) (top) or neoadjuvant chemotherapy (*n* = 49) (bottom). (B) Correlation between MCU expression levels and the palliative chemotherapy response (top) or the neoadjuvant chemotherapy response (bottom). (C) Representative IHC images of the MCU protein level in 129 PDAC tissues and paired adjacent non‐tumor tissues. Scale bar, 100 µm. The expression level is categorized as absent– (0), weak+ (1,2), moderate++ (3,4), and strong+++ (6,9). Heatmap showing a comparison of these protein levels between PDAC tissues (T) and paired adjacent non‐tumor tissues. (D) Kaplan–Meier curves showing OS or DFS in a cohort of 129 PDAC patients stratified by MCU protein levels. The log‐rank test *P‐*values are indicated. (E,F) MCU‐low and MCU‐high PDOs established from six PDAC patients were treated with a concentration gradient of AG regimen for 72 h. The radii of the PDOs were evaluated. PDX cells from the same patients were inoculated into NSG mice. Once the tumors reached a volume of approximately 100 mm^3^, they were subcutaneously transplanted into NSG mice and treated with AG 7 days later (defined as Day 0). Mice were humanely killed on Day 28. Representative images (E) and tumor inhibition rates (F) are shown. (G) Indicated cells were treated with varying concentrations of the AG regimen (a 20:1 molar ratio of gemcitabine to albumin‐bound paclitaxel; the labeled concentrations represent gemcitabine alone) for 72 h. The IC50 values were determined by the CCK‐8 assay. (H–K) Indicated cells were treated with 1000 nM AG for 48 h, and then cellular apoptosis was analyzed by flow cytometry. The representative dot plots (H and J) and statistical analysis (I and K) are shown. (L and M) Immunocompromised BALB/c nude mice were orthotopically inoculated with the indicated cells (*n* = 6 per group). The BLI intensity of tumors was monitored every 7 days by IVIS. Representative bioluminescent images of the mice at Day 28 after tumor inoculation and quantification of the radiance intensity (L and M) are shown. Representative tumor images and tumor inhibition rates of AG in the indicated groups at Day 28 (N and O) are shown. Quantification of TUNEL^+^ cells by IHC (P and Q). The tumor inhibition rate was calculated as: *(Volume_vehicle_‐Volume_AG‐treated_) /Volume_vehicle_*_._ Correlation analyses in (B) were performed using Pearson correlation analysis. Data in (C) were analyzed using the Wilcoxon signed‐rank test. Data in (J,K) were analyzed using two‐way ANOVA followed by Tukey's multiple comparison test. Data in (F), (I–K), and (N–Q) are presented as mean ± SD from 3 biological replicates and were analyzed using two‐sample, two‐tailed unpaired Student's *t‐*test. ns not significant, ^*^ *p* <0.05, ^**^ *p* <0.01, ^***^ *p* <0.001 and ^****^ *p* <0.0001. PR, partial remission; SD, stable disease; PD, progressive disease; NPD, non‐progressive disease; IVIS, in vivo imaging system. Scale bar, 100 µm.

To confirm the clinical relevance of MCU‐mediated chemoresistance, needle biopsies were collected from 68 patients with advanced PDAC who received AG‐based palliative chemotherapy. Immunohistochemistry (IHC) was used to assess MCU expression, and patients were stratified into MCU‐high and MCU‐low groups (Figure 2A). Patients in the MCU‐high group exhibited a significantly poorer response to palliative chemotherapy compared to those in the MCU‐low group (Figure 2B). In an independent cohort of 49 PDAC patients receiving AG‐based neoadjuvant chemotherapy, the MCU‐low group also showed a significantly higher objective response rate (ORR) than the MCU‐high group (Figure 2A,B). Additionally, MCU expression was significantly higher in tumor tissues than in paired adjacent non‐tumor tissues (Figure 2C). In a cohort of 129 PDAC patients who underwent radical R0 resection, patients with high MCU expression had significantly shorter overall survival (OS) and disease‐free survival (DFS) (Figure 2D). Univariate Cox proportional hazards analysis revealed that MCU expression was significantly associated with increased risks of death and tumor recurrence Table S1. Multivariate analysis further confirmed that MCU expression is an independent prognostic factor for both OS and DFS Table S1. Previous studies have demonstrated that MCU functions as the core subunit of the MCU complex, interacting with regulatory subunits (e.g., MICU1, MICU2, MICU3) that dynamically respond to extramitochondrial Ca^2+^ signals. In our cohort, while MICU1 and MICU2 expression showed a positive correlation with MCU in tumor tissues (Figure S2A), none of MICU1, MICU2, or MICU3 expression levels exhibited a significant prognostic association (Figure S2B–G). These findings were further validated using the TCGA dataset (Figure S2H–M).

Given that sensitivity to the AG regimen is a critical determinant of patient prognosis, we next investigated whether MCU promotes tumor progression by modulating AG chemoresistance. We established six patient‐derived xenografts (PDXs) and patient‐derived organoids (PDOs) from fresh PDAC tissues (Figure S2A). Both in vitro and in vivo experiments showed that MCU‐low PDXs and PDOs had significantly higher tumor inhibition rates after AG treatment compared to their MCU‐high counterparts (Figure 2E,F).

We then generated PDX‐derived cell lines with MCU overexpression (OE) or knockdown (KD). In vitro drug sensitivity assays revealed that the median inhibitory concentration (IC50) of AG was significantly elevated in MCU‐OE cells and reduced in MCU‐KD cells (Figure 2G). Following 48 h of treatment with 5 µM AG, MCU‐KD cells showed increased apoptosis rates, whereas MCU‐OE cells exhibited reduced apoptosis (Figure 2H–K). To further confirm these effects in vivo, we established orthotopic tumor models in BALB/c nude mice. Compared to the Scramble control group, MCU‐KD tumors displayed suppressed growth, enhanced responsiveness to AG, and higher proportions of TUNEL^+^ cells after AG administration (Figure 2L,N,P). In contrast, MCU‐OE tumors grew more rapidly, displayed diminished AG sensitivity, and had fewer apoptotic cells (Figure 2M,O,Q). Together, these data support a causative role for MCU in PDAC progression and chemoresistance.

### 3.3 MCU Facilitates Stemness Maintenance in PDAC

**FIGURE 3:**
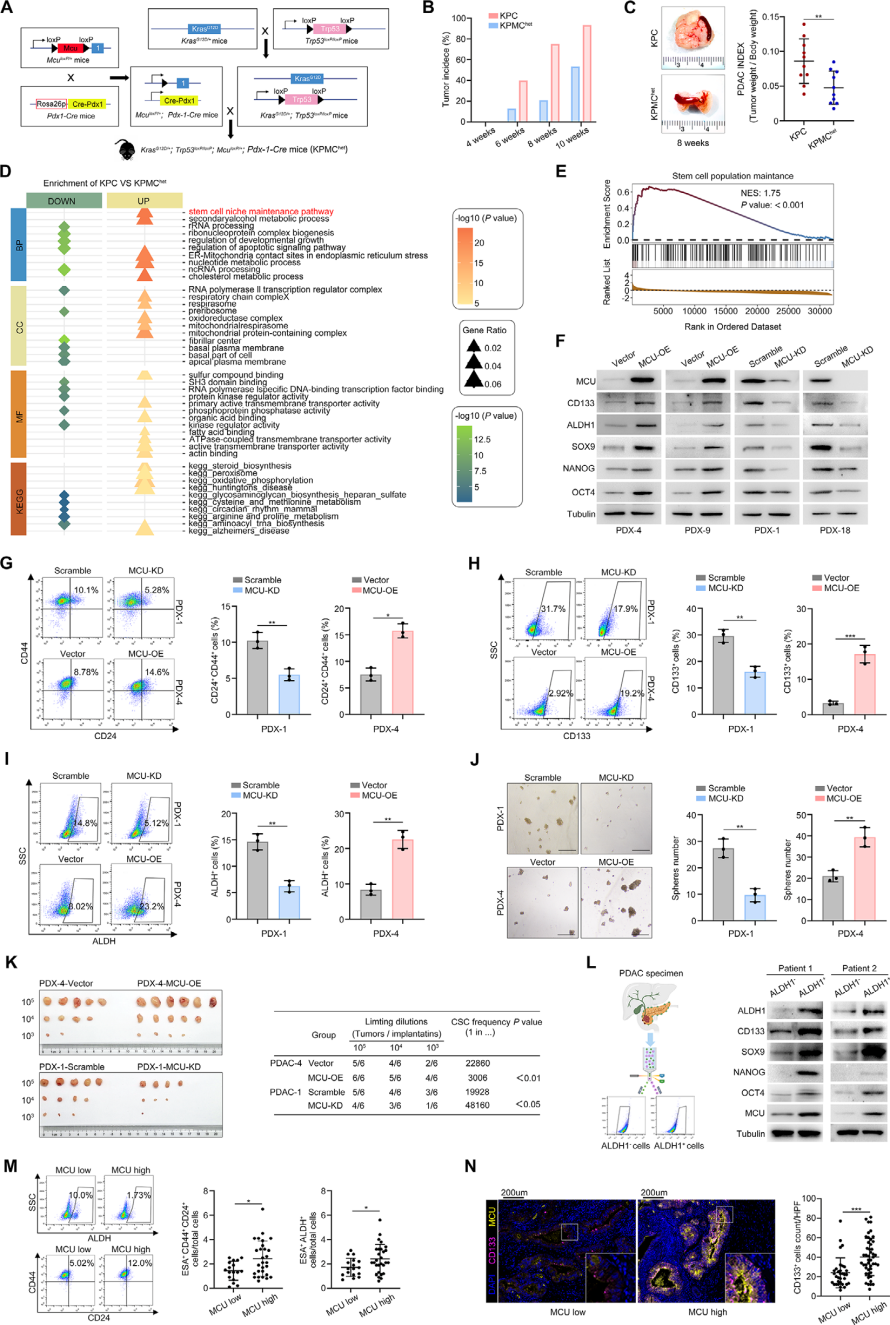
MCU plays a pivotal role in stemness maintenance in PDAC. (A) Schematic diagram showing the breeding strategy for conditioned KPMC^het^ mice. (B) Tumor incidence in KPC and KPMC^het^ mice at the indicated age. (C) Representative tumor images of 8‐week‐old KPC and KPMC^het^ mice and quantification of the tumor weight index. (D,E) Single‐cell RNA sequencing was performed in 8‐week‐old KPC (*n* = 4) and KPMC^het^ (*n* = 4) mice. GO pathway bubble plot showing DEGs in the epithelial cells of KPC mice vs. KPMC^het^ mice. Pathways are stratified by biological process (BP), cellular component (CC), and molecular function (MF) (D). GSEA enrichment score profile of the stem cell maintenance signal pathway between KPC and KPMC^het^ epithelial cells (E). (F) Western blots showing protein expression levels of MCU and stemness markers in the indicated cell lines. Representative results are shown. (G–I) Flow cytometry was used to determine the proportion of CD44^+^ CD24^+^ cells, CD133^+^ cells and ALDH^+^ cells in the indicated cell lines. Representative dot plots and percentage of CD44^+^ CD24^+^ cells (G), CD133^+^ cells (H), and ALDH^+^ cells (I) are shown. (J) Sphere formation assays were performed in the indicated cell lines. Representative images and quantification of sphere numbers are shown. Scale bar, 200 µm. (K) In vivo extreme limiting dilution assays were performed to assess the self­renewal capacity of the indicated cells. The probabilities of cancer stem cells are shown. (L) Schematic diagram showing the strategy for sorting ALDH^+^ epithelial cells and ALDH1^−^ epithelial cells from two fresh PDAC specimens. Western blots indicate that MCU expression levels are higher in ALDH^+^ cells than ALDH^−^ cells. (M) Single cell suspensions were prepared from 45 cases of fresh PDAC specimens and stained with ESA, CD24, CD44, or ALDEFLUOR. Representative dot plots and percentage of ALDH^+^ cells (E, left) and CD44^+^ CD24^+^ cells (gated on ESA^+^ epithelial cells) are shown. (N) mIHC staining of MCU and the stemness marker CD133 in 80 human PDAC tissues grouped by MCU expression. Scale bar, 200 µm. The proportions of CD133^+^ cells in the MCU‐high and MCU‐low groups are shown. Data in (C), (G–J), (M,N) are presented as mean ± SD and were analyzed using two‐sample, two‐tailed unpaired Student's *t‐*test. ns not significant, ^*^ *p* <0.05, ^**^ *p* <0.01, ^***^ *p* <0.001 and ^****^ *p* <0.0001.

To investigate the critical role of MCU in PDAC progression, we crossed *Kras^G12D/+^*; *Trp53^loxp/loxp^* mice with *Mcu^loxp/+^*; *Pdx‐1‐Cre* mice to generate a mouse model with pancreas‐specific heterozygous Mcu deletion on a background of spontaneous pancreatic tumorigenesis (termed KPMC^het^) (Figure 3A; Figure S3B,C). Compared to KPC controls, KPMC^het^ mice exhibited delayed tumor growth (Figure 3B) and reduced tumor weights at matched time points (Figure 3C). Single‐cell RNA sequencing of tumors from 8‐week‐old KPMC^het^ (*n* = 4) and KPC (*n* = 4) mice revealed significant enrichment of the stem cell niche maintenance pathway in epithelial cells (Figure 3D,E). The maintenance of cancer stemness facilitates the in situ progression of early‐stage PDAC and enables tumors to exhibit chemoresistance and metastatic properties [14]. Flow cytometry of primary epithelial cells demonstrated that downregulation of Mcu significantly reduced the proportion of Aldh^+^ tumor cells (Figure S3D), indicating that Mcu deficiency diminishes tumor stemness in KPC models.

We sought to verify similar trends in PDAC patients. Using six PDX‐derived cell lines, in vitro extreme limiting dilution assays confirmed a significant positive correlation between endogenous MCU expression and stem cell frequency (Figure S4A). Furthermore, MCU‐OE cells exhibited upregulated expression of stemness‐associated proteins (CD133, ALDH1, SOX9, NANOG, OCT4), whereas MCU knockdown significantly suppressed the expression of these proteins (Figure 3F). Flow cytometry confirmed that MCU overexpression significantly increased the proportions of CD44^+^ CD24^+^, CD133^+^, and ALDH^+^ cells (Figure 3G–I; Figure S4B–D), whereas MCU knockdown markedly diminished these stem cell populations. Further validation via in vitro spheroid formation assays showed that MCU enhanced the self‐renewal capacity of PDX cells, as reflected by a marked increase in spheroid number (Figure 3J; Figure S4E). In vivo extreme limiting dilution assays revealed higher stem cell frequency in MCU‐OE xenografts compared to the Vector control, while MCU knockdown reduced both parameters (Figure 3K). Additionally, we sorted ALDH^+^ cells from two fresh PDAC tumor tissues. Compared to ALDH^−^ cells, ALDH^+^ cells exhibited elevated MCU expression and enhanced stem‐like properties (Figure 3L). We further analyzed MCU expression levels and stem cell proportions in 36 fresh PDAC tumor tissues using IHC staining and flow cytometry. Notably, patients with high MCU expression had a significantly higher proportion of stem cells than those with low MCU expression (Figure 3M). Consistently, multiplex IHC staining in a separate cohort of 80 paraffin‐embedded PDAC tumor tissues revealed a positive correlation between MCU expression and the proportion of CD133^+^ tumor cells (Figure 3N).

Our previous work demonstrated that cancer stem cells (CSCs) are crucial mediators of chemotherapy resistance [15]. We therefore investigated whether stemness is a necessary requirement for MCU to promote AG resistance. To this end, we knocked down three key stemness‐maintaining transcription factors (SOX2, SOX9, and OCT4) in both MCU‐OE and Vector control cells (Figure S5A). Depletion of these factors completely abrogated the sphere formation capacity of MCU‐OE cells (Figure S5B). We then evaluated their response to AG treatment. Consistent with our hypothesis, triple knockdown of SOX2, SOX9, and OCT4 significantly attenuated MCU‐induced AG resistance both in vitro and in vivo (Figure S5C–F). Collectively, these findings indicate that MCU intrinsically maintains PDAC stemness, which likely contributes to its role in chemoresistance.

### 3.4 MCU Promotes Glutathione (GSH) Synthesis to Sustain PDAC Stemness

**FIGURE 4:**
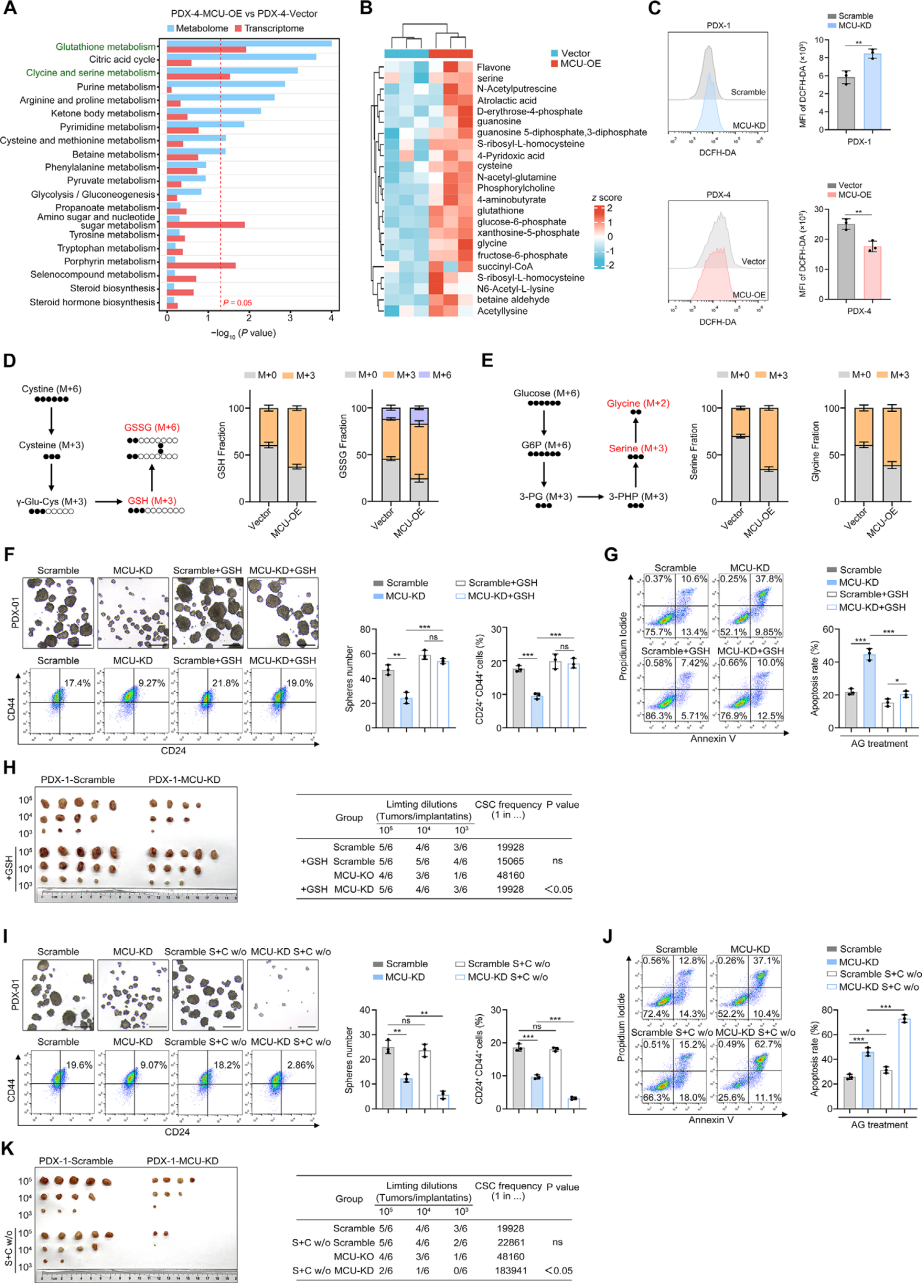
MCU regulates GSH synthesis and the de novo synthesis of serine. (A,B) Integrated metabolomic and transcriptomic analysis of MCU‐OE and Vector control PDX‐4 cells (A) Pathway enrichment analysis highlighting glutathione metabolism and glycine/serine metabolism as the most significantly perturbed pathways upon MCU overexpression at the metabolomic and/or transcriptomic level. (B) Heatmap depicting differentially abundant metabolites between groups. (C) Representative flow cytometry plots (left) and quantification of DCFH‐DA mean fluorescence intensity (MFI) (right) demonstrating the effects of MCU knockdown and overexpression on intracellular ROS levels. (D) Schematic showing ^13^C_6_,^15^N_2_‐cystine incorporation into GSH and GSSG. Incorporation of ^13^C and ^15^N into GSH and GSSG in PDX‐4 cells are shown. (E) Schematic showing ^13^C_6_‐glucose incorporation into serine and glycine. Incorporation of ^13^C into serine and glycine in PDX‐4 cells are shown. (F–H) The effects of exogenous GSH on MCU‐mediated stemness maintenance and chemoresistance. Sphere formation capacity (F), CD24^+^ CD44^+^ proportion (F), apoptotic rate after AG treatment (G), and self­renewal function (H) in PDX‐1‐MCU‐KD and PDX‐1‐Scramble control cells are shown. (I–K) The effects of serine and glycine deprivation on MCU‐mediated stemness maintenance and chemoresistance. Sphere formation capacity (I), CD24^+^ CD44^+^ proportion (I), apoptotic rate after AG treatment (J), and self­renewal function (K) in PDX‐1‐MCU‐KD and PDX‐1‐Scramble control cells are shown. Data in (C), (F,G), and (I,J) are presented as mean ± SD from 3 biological replicates and were analyzed using a two‐sample, two‐tailed unpaired Student's *t*‐test. ns not significant, ^*^ *p* <0.05, ^**^ *p* <0.01, ^***^ *p* <0.001 and ^****^ *p* <0.0001.

Mitochondrial Ca^2+^ signaling drives tumorigenesis by orchestrating energy metabolism, reactive oxygen species (ROS) homeostasis, and biosynthetic pathways [16, 17]. To elucidate how MCU enhances tumor stemness and chemoresistance, we performed integrated Kyoto Encyclopedia of Genes and Genomes (KEGG) enrichment analysis on combined metabolomic and transcriptomic data from MCU‐OE and Vector control PDX‐4 cells (Figure 4A). Pathway analysis revealed that glutathione metabolism, glycine and serine metabolism were the most significantly altered pathways at both metabolomic and transcriptomic levels. Notably, compared to Vector control cells, MCU‐OE cells exhibited elevated levels of glycine, serine, and cystine, which serve as key precursors for GSH biosynthesis (Figure 4B) [18, 19].

Given GSH's established role in ROS scavenging and stemness regulation [20, 21, 22], we hypothesized that MCU maintains stemness by preserving redox homeostasis. Consistent with this, MCU‐OE significantly reduced intracellular ROS levels and oxidative damage, whereas MCU‐KD increased both parameters (Figure 4C; Figure S6A). Exogenous supplementation with 1 mM N‐acetylcysteine (NAC) rescued MCU‐KD‐induced phenotypes, including elevated ROS and reduced stemness (Figure S6B–D). We quantified GSH metabolites via liquid chromatography–mass spectrometry (LC‐MS): in MCU‐OE cells, both intracellular GSH levels and the GSH:GSSG ratios were markedly increased (Figure S6E). NADPH, a critical reducing equivalent for maintaining GSH in its reduced state, also increased in MCU‐OE cells and decreased in MCU‐KD cells (Figure S6F,G).

To evaluate the dynamic synthesis rates of GSH and serine, we used ^13^C_6_,^15^N_2_‐cystine tracing and ^13^C_6_‐glucose tracing to assess GSH synthesis and serine/glycine synthesis. In Vector control cells, 40%–50% of the total GSH pool and 50%–60% of the total GSSG pool were labeled with ^13^C and ^15^N after 24 h; in MCU‐OE cells, the ^13^C and ^15^N incorporation into GSH and glycine increased to 50%–70% and 70%–80% (Figure 4D). For serine and glycine, 30%–40% of the total serine pool and 40%–50% of the total glycine pool in Vector control cells were labeled with ^13^C after 24‐h incubation, where as these labeling proportions increased to 60%–70% for both metabolites in MCU‐OE cells (Figure 4E). To confirm that GSH mediates MCU's effects on stemness, we supplemented the culture medium or diet of tumor‐bearing mice with exogenous GSH, the impairment of stemness and chemoresistance caused by MCU depletion were significantly rescued both in vitro and in vivo (Figure 4F–H). We also ruled out the involvement of the thioredoxin system, which is recognized as another key redox regulator. Western blot analysis revealed upregulated expression of TXNRD1 and TXN in MCU‐OE cells (Figure S6H). However, treatment with Auranofin, a thioredoxin system inhibitor, failed to reverse the MCU‐OE‐induced increases in sphere formation capacity and IC50 values of AG (Figure S6I,J), indicating that the effects of MCU are primarily dependent on GSH biosynthesis.

Given that MCU deficiency inhibits *de novo* serine and glycine synthesis, we hypothesized that serine/glycine deprivation would further suppress GSH biosynthesis. As expected, serine/glycine deprivation in MCU‐KD cells markedly reduced stemness and chemoresistance, whereas minimal effects were observed in Scramble control cells (Figure 4I–K). In summary, our data demonstrate that MCU promotes the *de novo* serine and glycine synthesis in PDAC, which sustains GSH production and thereby alleviates ROS‐mediated inhibition of stemness.

### 3.5 MCU Promotes GSH Synthesis via PSAT1 and SLC7A11

**FIGURE 5:**
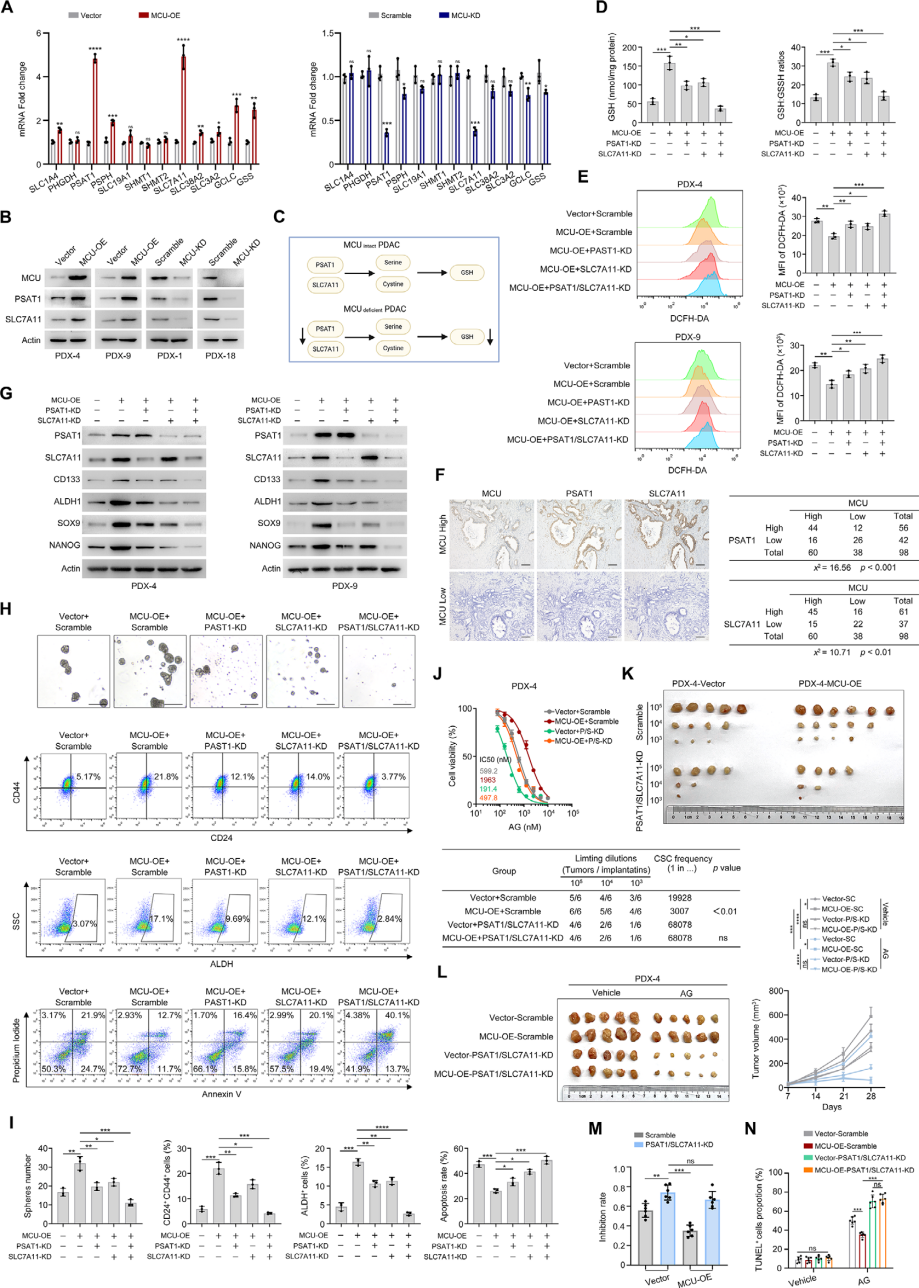
PSAT1 and SLC7A11 confer PDAC stemness and chemoresistance. (A) qPCR analysis showing the effects of MCU overexpression and knockdown on the expression levels of the indicated metabolic enzymes. (B) Western blot analysis showing the effects of MCU overexpression and knockdown on PSAT1 and SLC7A11 protein expression in PDX cells. (C) Schematic diagram showing the potential regulation of GSH synthesis by MCU via PSAT1 and SLC7A11. (D) The effects of SLC7A11 knockdown and knockdown on intracellular GSH levels and GSH:GSSG ratios in PDX‐4 cells. (E) Representative flow cytometry images and quantitation of DCFH‐DA mean fluorescence intensity showing the effects of SLC7A11 knockdown and PSAT1 knockdown on ROS levels in the indicated cells. (F) IHC staining of MCU, PSAT1, and SLC7A11 expression in PDAC sections. Correlation analysis of these proteins in 98 PDAC cases is quantified. Sequential sections were used for the IHC staining. (G) Western blot analysis showing the effects of SLC7A11 knockdown and PSAT1 knockdown on MCU‐mediated stemness marker expression levels in the indicated cell lines. (H,I) The effects of SLC7A11 knockdown and PSAT1 knockdown on MCU‐mediated stemness maintenance and chemoresistance in vitro. Sphere formation capacity, CD24^+^ CD44^+^ proportion, ALDH^+^ proportion, and apoptotic rate following AG treatment of PDX‐4 cells in the indicated groups are shown. Scale bar, 100 µm. (J) Dose response curves showing the effects of SLC7A11 and PSAT1 double knockdown on sensitivities to AG treatment in the indicated cells. (K) In vivo extreme limiting dilution assays showing the effects of SLC7A11 and PSAT1 double knockdown on the self­renewal ability of the indicated cells. The probabilities of cancer stem cells are shown. (L–N) The effects of SLC7A11 and PSAT1 double knockdown on chemoresistance in vitro. Immunocompromised BALB/c nude mice were subcutaneously inoculated with the indicated cells (*n* = 6 per group). Mice were administered vehicle or AG 7 days later (defined as Day 0) and humanely killed on Day 28. Representative tumor images and growth curves (L), inhibition rates (M), and the quantification of TUNEL^+^ cells by IHC (N) are shown. All experiments were repeated three times independently. Data in (L) were analyzed using two‐way ANOVA followed by Tukey's multiple comparison test. Data in (A), (D,E), (I,J), and (M,N) are presented as mean ± SD from 3 biological replicates and were analyzed using a two‐sample, two‐tailed unpaired Student's *t*‐test. ns not significant, ^*^ *p* <0.05, ^**^ *p* <0.01, ^***^ *p* <0.001 and ^****^ *p* <0.0001.

The aberrant expression of key metabolic enzymes extensively alters metabolic flux, thereby driving metabolic reprogramming to support tumor progression [23, 24, 25]. Here, we focused on key enzyme‐coding genes involved in *de novo* serine synthesis, cystine transport, and GSH biosynthesis. qPCR results analysis revealed that among the panel of genes examined, the expression levels of PSAT1 and SLC7A11 were significantly upregulated in MCU‐OE cells; consistently, their expression was also reduced in MCU‐KD cells (Figure 5A). Functionally, PSAT1 governs *de novo* serine synthesis, which serves as a critical metabolic hub to fuel one‐carbon metabolism, nucleotide production, and GSH biosynthesis [20, 26, 27]. SLC7A11 regulates cystine transport across the plasma membrane. Western blot analysis showed that MCU overexpression increased the protein levels of PSAT1 and SLC7A11 in PDX cells, whereas MCU knockdown reduced their expression (Figure 5B). Based on these findings, we hypothesized that PSAT1 and SLC7A11 cooperatively drive GSH biosynthesis under MCU regulation (Figure 5C).

To test this hypothesis, we performed individual or combined knockdown of PSAT1 and SLC7A11 in MCU‐OE cells. As shown in Figure 5D, Individual knockdown of either PSAT1 or SLC7A11 partially reversed the MCU‐OE‐induced increases in intracellular GSH levels and the GSH:GSSG ratio. Notably, dual knockdown of PSAT1 and SLC7A11 completely abrogated the MCU‐driven GSH metabolism reprogramming. A consistent trend was observed for intracellular ROS levels: individual gene knockdown partially reduced MCU‐OE‐induced ROS production, while dual knockdown fully restored ROS levels (Figure 5E). To extend these findings to clinical samples, we performed IHC staining of 98 PDAC patient specimens. This analysis further confirmed significant positive correlations between MCU expression and the protein levels of both PSAT1 and SLC7A11 (Figure 5F). We next investigated whether PSAT1 and SLC7A11 mediate the effects of MCU on stemness and chemoresistance. Individual knockdown of PSAT1 or SLC7A11 partially restored the expression of stemness markers, spheroid formation capacity, CSC ratios, and chemosensitivity in MCU‐OE cells. In contrast, dual knockdown of PSAT1 and SLC7A11 nearly fully reversed these phenotypes (Figure 5G–J; Figure S7A,B). To validate these results in vivo, we performed a limited dilution assay, which demonstrated that MCU overexpression significantly increased the stem cell frequency. This increase was abrogated following the dual knockdown of PSAT1 and SLC7A11 (Figure 5K). As anticipated, the dual knockdown also markedly inhibited the resistance to AG induced by MCU in PDAC cells (Figure 5L–N). Collectively, these data establish that MCU upregulates PSAT1 and SLC7A11, which synergistically drive GSH biosynthesis. This metabolic reprogramming promotes stemness and chemoresistance in PDAC.

### 3.6 MCU Drives Upregulation of PSAT1 and SLC7A11 Through the ER‐PERK Axis in PDAC

**FIGURE 6:**
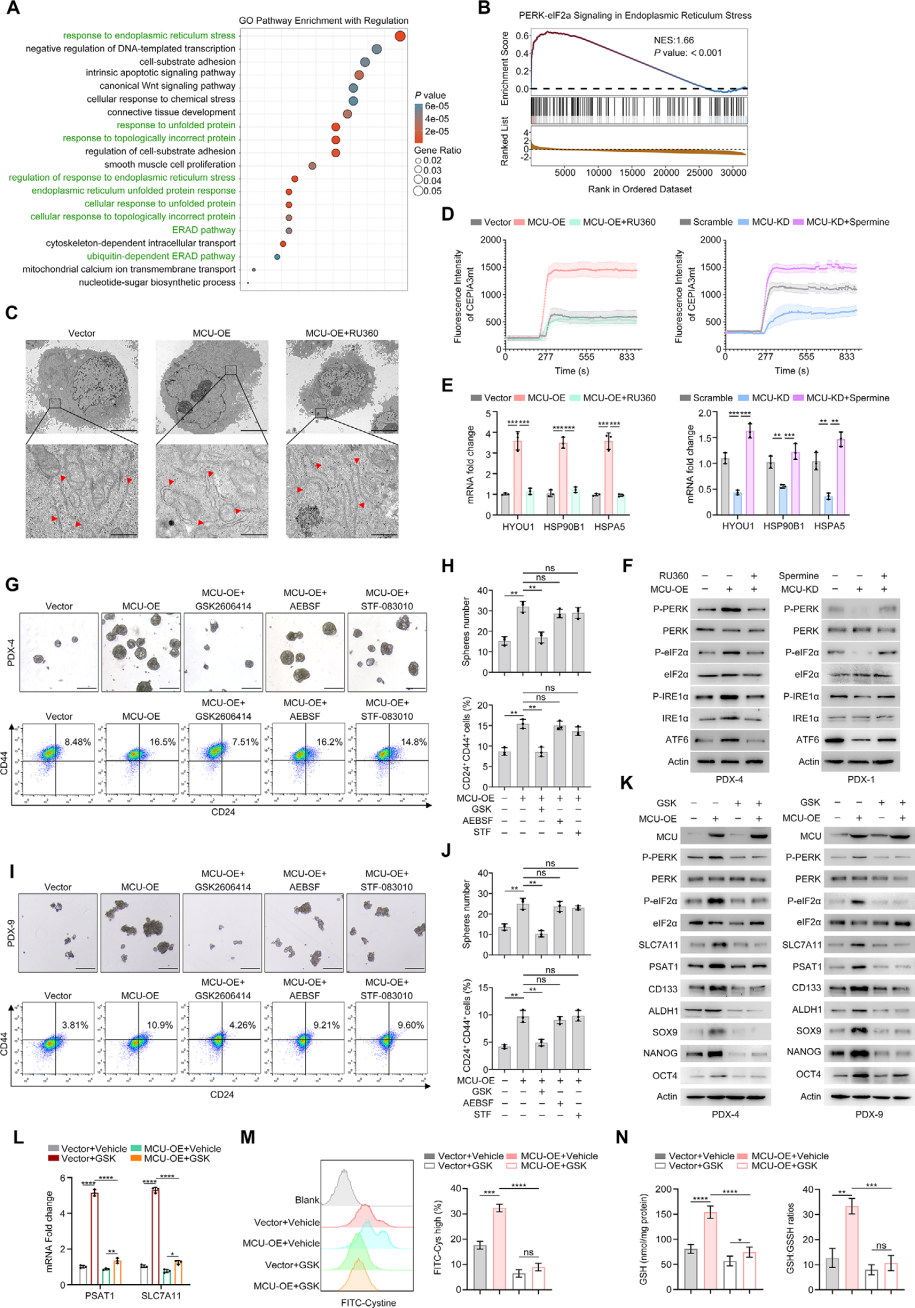
MCU mediates ER stress‐inducing metabolic reprogramming and stemness maintenance. (A) GO pathway enrichment analysis was used to identify the differential signaling pathways between MCU‐OE and Vector control PDX‐4 cells in transcriptome sequencing. (B) Gene Set Enrichment Analysis (GSEA) enrichment score profile of (A). (C) Representative electron microscopy images showing ER morphological changes (yellow arrow) in the indicated cell lines. Scale bar, 2 µm. (D) The effects of RU360 and spermine treatment on MCU‐mediated mitochondrial Ca^2+^ influx in PDX‐4 and PDX‐1 cells. (E) qPCR analysis showing the effects of MCU overexpression and MCU knockdown on ER stress‐related gene expression levels in PDX‐4 and PDX‐1 cells. (F) Western blots showing the effects of MCU overexpression and MCU knockdown on the activation of three downstream canonical UPR branches: PERK, ΙRΕ1α, and ATF6, in the indicated cell lines. (G–J) The effects of pharmacological inhibition of each of the three downstream targets on MCU‐mediated stemness maintenance in vitro. Representative images of (G–I) and quantification (H–J) of sphere formation capacity and CD24^+^ CD44^+^ proportion of PDX‐4 and PDX‐9 cells in the indicated groups. Scale bar, 100 µm. (K) Western blots showing the effects of the PERK inhibitor GSK2606414 on MCU‐mediated stemness marker expression levels in the indicated cells. (L) qPCR analysis showing the effects of GSK2606414 on PSAT1 and SLC7A11 mRNA expression levels in the indicated cells. (M) Flow cytometry was used to determine the effects of GSK2606414 on the uptake of FITC‐cystine in PDX‐4 cells. (N) The effects of GSK2606414 on intracellular GSH levels and GSH:GSSG ratios in PDX‐4 cells. Data in (E), (G–J), and (L–N) are presented as mean ± SD from 3 biological replicates and were analyzed using two‐sample, two‐tailed unpaired Student's *t*‐test. ns not significant, ^*^ *p* <0.05, ^**^ *p* <0.01, ^***^ *p* <0.001 and ^****^ *p* <0.0001.

To elucidate the underlying mechanism by which MCU regulates PSAT1 and SLC7A11, we performed Gene Ontology (GO) enrichment analysis on the transcriptomic sequencing data, with a specific focus on pathway‐related changes in biological processes (BP). The results revealed that Endoplasmic Reticulum stress‐related pathways were significantly upregulated in MCU‐OE cells (Figure 6A). AS the ER and mitochondria are tightly connected via mitochondria‐associated ER membranes (MAMs), dysregulation of Ca^2+^ crosstalk between these two organelles leads to the accumulation of unfolded proteins in the ER, thereby triggering ER stress and downstream cascading reactions [28, 29]. Furthermore, Gene Set Enrichment Analysis (GSEA) validated a strong association between MCU overexpression and the activation of the PERK‐elF2a signaling in Endoplasmic Reticulum stress, a key component of the unfolded protein response (UPR) (Figure 6B). Using organelle‐targeted Ca^2^^+^ sensors, we found that MCU overexpression was sufficient to promote ER Ca^2^^+^ efflux and mitochondrial Ca^2^^+^ uptake, whereas knockdown of MCU inhibits the transfer of Ca^2^^+^ from the ER to mitochondria. (Figure S8A–C). Similarly, we used the MCU inhibitor RU360 and the MCU agonist spermine to mimic the changes in Ca^2+^ influx caused by altered MCU expression. Ultrastructural analysis via transmission electron microscopy revealed that MCU overexpression induced ER expansion, which was characterized by dilated cisternae and fragmented rough ER membranes; this phenotype was reversed by RU360 treatment (Figure 6C). Likewise, we found that MCU increased the expression of heat shock proteins and activated related downstream pathways in a Ca^2^^+^ uptake‐dependent manner (Figure 6D–F).

It has been reported that ER stress has been reported to maintain cellular homeostasis by mediating the adaptive expression of three downstream canonical UPR branches (PERK, ATF6, and IRE1α). To delineate its role in MCU‐mediated stemness enhancement, MCU‐OE cells were treated with specific inhibitors targeting each UPR branch: GSK2606414 (PERK inhibitor), AEBSF (ATF6 inhibitor), and STF‐083010 (IRE1α inhibitor). Notably, PERK inhibition by GSK2606414 significantly restored sphere formation capacity and the stemness ratio of MCU‐OE cells, whereas ATF6 or IRE1α blockade had negligible effects. These results establish the PERK signaling as primarily mediating MCU‐driven stemness (Figure 6G–J). pharmacological inhibition of the PERK‐eIF2α pathway in MCU‐OE cells not only normalized stemness marker expression levels but also restored the expression of PSAT1 and SLC7A11 to their baseline levels (Figure 6K,L). Concurrently, following GSK2606414 treatment, both cystine uptake and GSH biosynthesis were markedly suppressed in MCU‐OE cells (Figure 6M,N). Collectively, these findings demonstrate that MCU activates PERK in a Ca^2^^+^‐dependent manner which in turn upregulates the expression of PSAT1 and SLC7A11. This cascade ultimately enhances GSH production and sustains PDAC stemness.

### 3.7 MCU Upregulates PSAT1 and SLC7A11 Expression in an ATF4‐ and NRF2‐Dependent Manner

**FIGURE 7:**
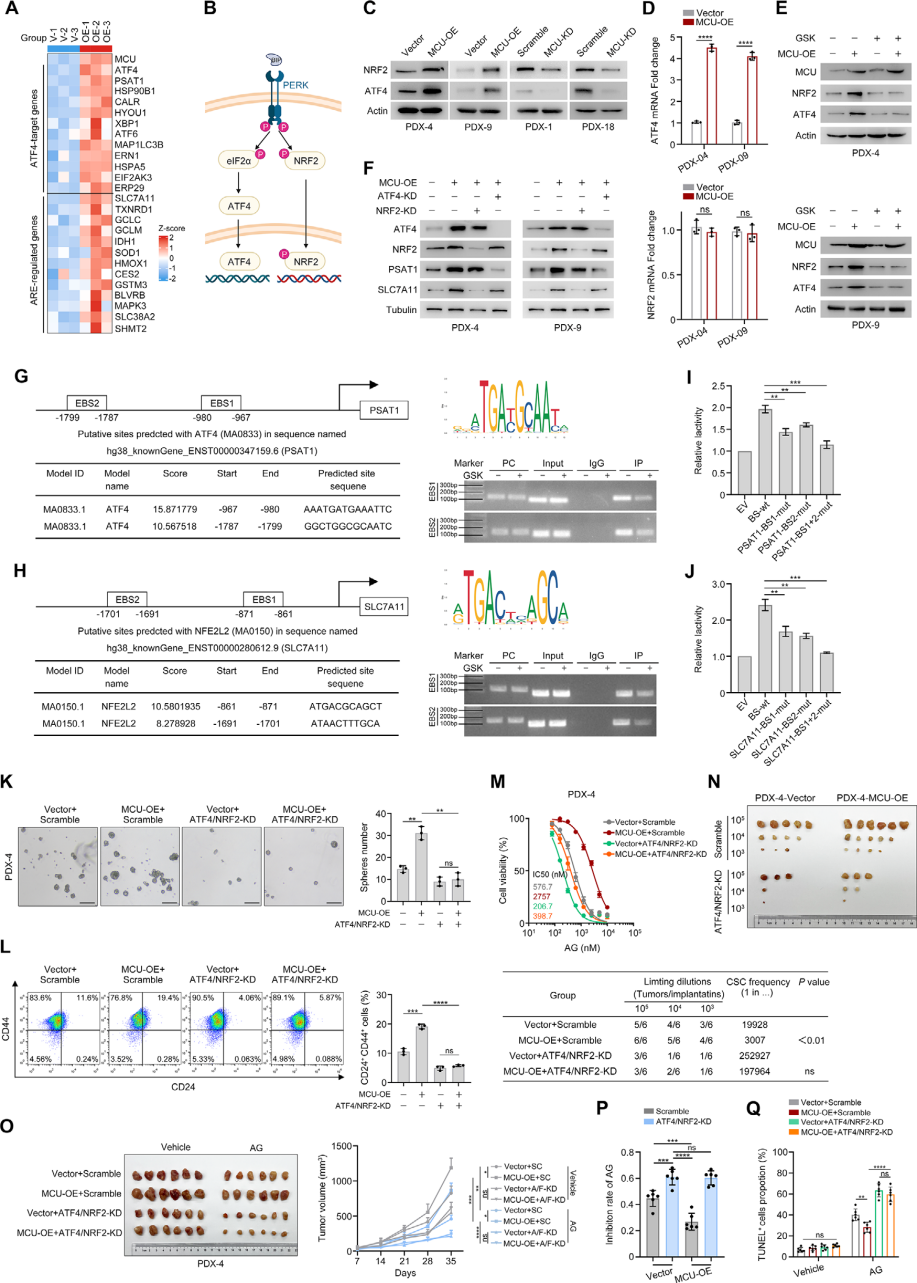
MCU upregulates the ATF4‐PSAT1 and NRF2‐SLC7A11 axis via the PERK‐eIF2a signaling pathway. (A) Heatmap showing the differential expression of ATF4‐target genes and ARE‐regulated genes in MCU‐OE and Vector control PDX‐4 cells. (B) Schematic diagram of the PERK‐eIF2α signaling pathway. (C) Western blots showing the effects of MCU overexpression and MCU knockdown on NRF2 and ATF4 expression levels in PDX cells. (D) mRNA transcript levels of ATF4 and NRF2 in MCU‐OE and Vector control PDX cells. (E) Western blots showing the effects of GSK2606414 on NRF2 and ATF4 expression levels in the indicated cells. (F) Western blots showing the effects of ATF4 knockdown and NRF2 knockdown on MCU‐mediated PSAT1 and SLC7A11 protein expression levels. (G,H) The ChIP assay was used to confirm the binding of ATF4 (G) to the PSAT1 promoter and NRF2 (H) to the SLC7A11 promoter in PDX‐4 cells. (I,J) The dual‐luciferase reporter assay was performed to determine the promoter activity in PDX cells. Renilla luciferase activity was used as the internal control. (K,L) The effects of ATF4 and NRF2 double knockdown on MCU‐mediated stemness maintenance in vitro. Sphere formation capacity (K) and CD24^+^ CD44^+^ proportion (L) of PDX‐4 cells in the indicated groups are shown. Scale bar, 100 µm. (M) Dose response curves showing the effects of ATF4 and NRF2 double knockdown on sensitivities to AG treatment in the indicated cells. (N) In vivo extreme limiting dilution assays showing the effects of ATF4 and NRF2 double knockdown on the self­renewal ability of the indicated cells. The probabilities of cancer stem cells are shown. (O–Q) The effects of ATF4 and NRF2 double knockdown on chemoresistance in vivo. Immunocompromised BALB/c nude mice were subcutaneously inoculated with the indicated cells (*n* = 6 per group). Mice were administered vehicle or AG 7 days later (defined as Day 0) and humanely killed on Day 35. Representative tumor images and growth curves (O), inhibition rates (P), and the quantification of TUNEL^+^ cells by IHC (Q) are shown. Data in (O) were analyzed using two‐way ANOVA followed by Tukey's multiple comparison test. Data in (D), (I–L), and (P,Q) are presented as mean ± SD from 3 biological replicates and were analyzed using two‐sample, two‐tailed unpaired Student's *t*‐test. ns not significant, ^*^ *p* <0.05, ^**^ *p* <0.01, ^***^ *p* <0.001 and ^****^ *p* <0.0001.

Among the differentially expressed genes (DEGs) in MCU‐OE cells, besides PSAT1 and SLC7A11, we also identified multiple classical ATF4‐target genes, as well as NRF2‐target genes that harbor the antioxidant response element (ARE) (Figure 7A). Beyond its role in phosphorylating eIF2α, PERK can further directly phosphorylate NRF2 and promote its nuclear translocation [26, 30] (Figure 7B). To verify whether MCU regulates NRF2 and ATF4, we performed Western blot and qPCR analyses which revealed that MCU overexpression increased the protein levels of both ATF4 and NRF2 (Figure 7C). At the transcriptional level, MCU overexpression elevated ATF4 mRNA levels but had no significant effect on NRF2 mRNA expression (Figure 7D). We further found that when MCU‐OE cells were treated with the PERK inhibitor GSK2606414, MCU‐induced upregulation of ATF4 and NRF2 was significantly suppressed, even though Ca^2+^ influx remained enhanced (Figure 7E; Figure S9A). Additionally, we used short‐term spermine treatment to mimic the effect of MCU overexpression; this treatment activated ATF4 and NRF2, and the effect was also blocked by GSK2606414 (Figure S9B,C). These results suggest a PERK‐dependent activation mechanism.

To determine whether ATF4 and NRF2 transcriptionally activate PSAT1 and SLC7A11, we performed genetic knockdown experiments. In MCU‐OE cells, ATF4 knockdown significantly reduced both the protein and mRNA levels of PSAT1, while NRF2 knockdown similarly led to a decrease in the protein and mRNA levels of SLC7A11(Figure 7F; Figure S9D). Then we predicted two ATF4‐binding motifs in the PSAT1 promoter and two NRF2‐binding sites in the SLC7A11 promoter using the JASPAR database. Chromatin immunoprecipitation (ChIP) assays confirmed robust ATF4 occupancy at the PSAT1 promoter (Figure 7G) and NRF2 binding to the SLC7A11 promoter (Figure 7H), whereas GSK2606414 treatment reducing promoter occupancy. Dual‐luciferase reporter assays using promoter mutants deficient in ATF4/NRF2 motifs revealed that each binding site was important for full transcriptional activity, as double mutations abolished the luciferase signals (Figure 7I,J).

We then evaluated whether ATF4 and NRF2 activation are essential. In vitro experiments were performed using ATF4 or NRF2 Knockdown in MCU‐OE cells, revealing that individual knockdown of either factor only partially reversed MCU‐induced phenotypic changes (Figure S9E–J). Double knockdown of ATF4 and NRF2 significantly restored tumor sphere formation capacity (Figure 7K), ALDH1^+^ proportion (Figure 7L), and IC50 values of AG (Figure 7M) in MCU‐OE cells. We then performed in vivo extreme limiting dilution assays and drug administration experiment in tumor‐bearing mice and observed similar results, in that MCU‐induced upregulation of stem cell frequency (Figure 7N) and chemoresistance (Figure 7O–Q) were fully abrogated. Collectively, these findings demonstrate that PERK‐dependent ATF4/NRF2 activation primarily mediates MCU‐induced PSAT1/SLC7A11 expression as well as downstream GSH homeostasis and stemness.

### 3.8 High‐Throughput Screening and Target Specificity Profiling of MCU Inhibitors

We next investigated the translational potential of targeting MCU. MCU knockout downregulated the expression of regulatory subunits EMRE, MICU1, and MICU2, resulting in chronic impairment of mitochondrial Ca^2+^ uptake capacity (Figure S10A–B). Although other transporters and regulators of mitochondrial function (e.g., Letm1, UCP2) have been reported to modulate mitochondrial Ca^2+^ uptake in specific cell types or under metabolic stress, no compensatory upregulation of these factors was detected in MCU‐KO cells (Figure S10B). Furthermore, the expression of the Na^+^/Ca^2^^+^ exchanger (NCLX) was unaltered, and its encoding gene SLC8A1 exhibited no significant correlation with the prognosis of PDAC patients (Figure S10C). These findings indicate that strategies targeting MCU exhibit both efficacy and stability.

MCU function is crucial in normal cells (e.g., neurons, cardiomyocytes), and systemic inhibition of its activity may thus induce significant toxic side effects. Therefore, we proposed a strategy targeting MCU upstream regulators in PDAC. First, SCENIC (Single‐Cell Regulatory Network Inference and Clustering) was utilized to identify PDAC cell type‐specific MCU regulons, revealing that RCOR1 was among the top‐ranked regulons (Figure S11A). We knocked down RCOR1 in PDX cells and confirmed its potential regulatory role in regulating MCU expression (Figure S11B). By analyzing the crystal structure of RCOR1 and key amino acid residues involved in active site binding, we performed virtual screening to identify small‐molecule compounds capable of competitively binding to these domains and potentially inhibiting MCU expression (Figure S11C,D). Approximately 400,000 RCOR1‐targeting compounds from the CHEMBL library were initially screened and filtered. High‐throughput virtual screening (HTVS) and standard precision (SP) docking were performed on the natural product libraries L4000 and L6020, followed by molecular docking and protein‐ligand interaction fingerprint (PLIF) analysis. Clinical safety was further evaluated by absorption, distribution, metabolism, and excretion (ADME) profiling to finalize candidate molecules. A total of 313 RCOR1‐targeting compounds with high binding affinity and low toxicity were identified, among which NB‐598 stood out (Figure S11E). Notably, treatment with NB‐598 from a functional small‐molecule drug library significantly reduced MCU expression and sensitized PDAC cells to AG treatment (Figure S11F–H). To elucidate the molecular basis of NB‐598 binding to human RCOR1, we visualized the interaction of NB‐598 with the RCOR1 active sites and delineated the specific interactions between amino acid residues within these sites (Figure S11I). Cellular Thermal Shift Assay (CETSA) also provided evidence that NB‐598 treatment enhanced the thermal stability of RCOR1, consistent with the high binding affinity predicted by virtual screening (Figure S11J).

**FIGURE 8:**
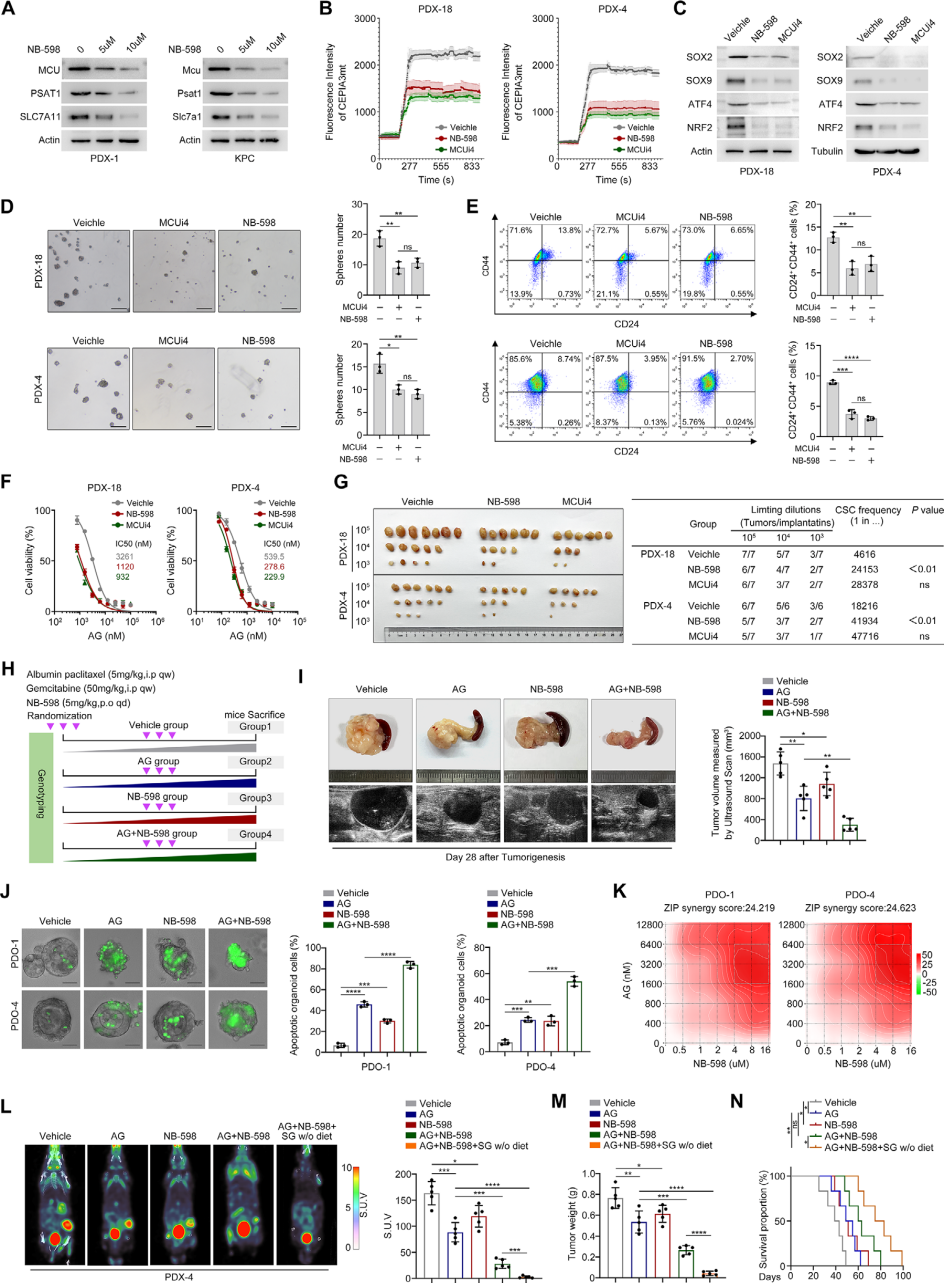
Targeting MCU in PDAC increases the efficacy of AG. (A) Western blots showing the effects of NB‐598 on the expression levels of MCU, PSAT1, and SLC7A11 in PDX‐1 and KPC cells. (B) The effects of NB‐598 and the classic MCU inhibitor MCUi4 on mitochondrial Ca^2+^ influx. PDX‐18 cells were treated with 10 µM NB‐598 or 10 µM MCUi4 for 24 h (left). PDX‐4 were cells treated with 10 µM NB‐598 or 5 µM MCUi4 for 24 h (right). (C) Western blots showing the effects of NB‐598 and MCUi4 on the expression levels of SOX2, SOX9, ATF4, and NRF2 in PDX‐18 and PDX‐4 cells. (D,E) The effects of NB‐598 and MCUi4 on stemness maintenance in vitro. Sphere formation capacity (D), CD24^+^ CD44^+^ proportion (E) in PDX‐18 and PDX‐4 cells are shown. Scale bar, 100 µm. (F) Dose response curves showing the effects of NB‐598 and MCUi4 on sensitivities to AG treatment in PDX‐18 and PDX‐4 cells. (G) In vivo extreme limiting dilution assays showing the effects of NB‐598 and MCUi4 on the self­renewal ability of PDX‐18 and PDX‐4 cells. The probabilities of cancer stem cells are shown. (H) Schematic illustration showing the experimental design. Genetically engineered KPC mice were randomized into four groups (*n* = 6 per group): (A) vehicle, (B) AG, (C) NB‐598, and (D) AG + NB‐598 (Albumin paclitaxel: 5 mg/kg, i.p., qw; Gemcitabine: 50 mg/kg, i.p., qw; and NB‐598: 5 mg/kg, p.o., qd). (I) Representative ultrasonic images and tumor images of KPC mice in the indicated groups at day 28 after tumorigenesis (left). Tumor volumes are shown (right). (J,K) PDO‐1 and PDO‐2 were pre‐treated with DMSO or NB‐598 for 24 h, then treated with DMSO or AG for a further 24 h. PDAC organoid apoptosis was examined at constant concentrations (NB‐598: 5 µM and AG: 10 µM) using caspase 3/7 green fluorescence reagent (J) or at different concentrations for analyzing the synergy score of NB‐598 and AG using Synergy finder (K). Scale bar, 20 µm. (L‐M) PDX‐1 tumors were transplanted into NSG mice and randomized into five groups (*n* = 6 per group): (A) vehicle, (B) AG, (C) NB‐598, (D) AG + NB‐598, and (E) AG + NB‐598 + SG w/o diet. Drugs were administered 7 days later (defined as Day 0) and mice were humanely killed on Day 28. Mice were subjected to PET‐CT to evaluate tumor burden after 3 weeks. Representative images of PET‐CT scans and the statistical analyses of tumor SUV of each group at Day 28 (L) are shown. Tumor weights of the indicated groups are shown in (M). (N) Immunocompromised BALB/C nude mice were orthotopically inoculated with PDX‐1 cells (*n* = 6 per group). Kaplan–Meier survival curves of mice in the indicated groups based on the log‐rank statistic test are shown. Data in (D,E), (I,J), and (L,M) are presented as mean ± SD from 3 biological replicates and were analyzed using a two‐sample, two‐tailed unpaired Student's *t*‐test. ns not significant, ^*^ *p* <0.05, ^**^ *p* <0.01, ^***^ *p* <0.001 and ^****^ *p* <0.0001.

Next, we investigated the targeting specificity of NB‐598 for MCU inhibition, with the established MCU inhibitor MCUi4 as a positive control in comparative inhibition assays. NB‐598 treatment significantly reduced the expression of MCU and its downstream metabolic enzymes (Figure 8A). Mitochondrial Ca^2+^ influx assays revealed that 10 µM NB‐598 inhibited Ca^2+^ influx in PDX‐18 cells, with efficacy comparable to 10 µM MCUi4; in PDX‐4 cells, 10 µM NB‐598 exerted an inhibitory effect on Ca^2+^ influx similar to that of 5 µM MCUi4 (Figure 8B). At optimized doses, NB‐598 exhibited inhibitory effects on MCU and its downstream phenotypes comparable to those of MCUi4 (Figure 8C–G). Furthermore, when NB‐598 was administered to RCOR1‐KD cells, its inhibitory effects on MCU expression (Figure S11K), and on MCU‐mediated stemness maintenance and chemoresistance (Figure S11L,M) were markedly attenuated. Similarly, NB‐598 showed significantly reduced inhibition of stemness maintenance and chemoresistance in MCU‐KO cells (Figure S11N,O). These results preliminarily validate that NB‐598 specifically targets and inhibits MCU through RCOR1 thereby further suppressing the MCU downstream signaling pathways associated with stemness maintenance.

### 3.9 NB‐598 Demonstrates Favorable Tolerability and Exerts a Synergistic Sensitizing Effect In Vivo

In the nude mouse model, we assessed the efficacy and toxicity profile of combined therapy with NB‐598 and AG at safe doses, using MCUi4 (administered at the previously reported dose) for comparison. Results showed that NB‐598 exhibited tumor‐suppressive effects and chemosensitizing effects comparable to those of MCUi4 (Figure S12A–C). Moreover, when combined with the AG regimen, relative to MCUi4, NB‐598 had a less significant impact on mouse weight loss (Figure S12D). At Day 28, no detectable changes in hematological parameters of RBC, WBC, platelet count, and hemoglobin among the groups (Figure S13A). Elevated biochemical parameters (AST and ALT) were detected exclusively in the MCUi4 combined AG group, while the liver function of other groups is normal (Figure S13A). In addition, there were no significant differences in biochemical parameters related to renal function (creatinine and BUN) and myocardial function (LDH) across all groups (Figure S13A). Then the mice were sacrificed, and H&E staining assays were performed on the stomach, duodenum, colon, lung, kidney, liver, and heart tissues. The results revealed that vacuolar degeneration of the liver was observed in the MCUi4 combined with the AG group, while no significant damage was detected in other organs across all groups (Figure S14A). Collectively, these findings preliminarily validate the safety and efficacy of the NB‐598 combined AG treatment in vivo.

We then examined the effects of AG and NB‐598 treatment in the genetically engineered KPC mouse model of PDAC. PDAC tumor formation in KPC mice was monitored by ultrasound imaging. Mice were randomly assigned to one of four treatment groups (vehicle, AG, NB‐598, or combination) when tumor volume reached ∼50 mm^3^ (Figure 8H). After four weeks of treatment, AG and NB‐598 reduced KPC tumor volumes by 43% and 29%, respectively, while combination treatment achieved a 79% reduction in tumor size (Figure 8I), consistent with our observations in orthotopic tumor models. In the PDO model, we administered NB‐598 with AG and measured caspase 3/7 activation levels as an indicator of apoptosis. As expected, NB‐598 sensitized PDOs to AG‐induced apoptosis (Figure 8J), with dose‐dependent synergistic effects (Figure 8K). Additionally, we used NSG mouse models bearing PDX tumors to determine whether dietary deprivation of serine and glycine could enhance chemosensitivity. The inhibitory effects of monotherapy and combination therapy on tumor growth were further validated by positron emission tomography using ^18^F‐deoxyglucose as a tracer (Figure 8L). We found that MCU inhibition combined with serine and glycine dietary deprivation further increased tumor sensitivity to AG treatment (Figure 8M). Ki67 staining of tumor tissues from the indicated treatment groups also demonstrated that combination treatment significantly suppressed tumor proliferation (Figure S15A). Finally, we validated the survival benefits of this combination strategy using an orthotopic tumor model. Our results showed that NB‐598 combined with AG and serine and glycine dietary deprivation significantly prolonged survival in tumor‐bearing mice (Figure 8N). Collectively, these findings suggest that targeting the MCU‐serine‐GSH axis provides a potential approach for PDAC treatment.

## 4 Discussion

Gemcitabine‐based chemotherapy remains the first‐line therapy for PDAC at all clinical stages [29]. While these regimens effectively target proliferating tumor cells, they consistently fail to eradicate quiescent stem‐like tumor cells, a therapy‐resistant subpopulation that drives chemoresistance through enhanced DNA repair, upregulated drug efflux pumps, and activation of anti‐apoptotic signaling pathways [31, 32, 33]. Thus, innovative strategies to selectively target stem‐like tumor cells are imperative to address this critical therapeutic gap [8]. In this study, through integrative multi‐omics analyses and validation across multiple models, we identified MCU as a promising regulator of tumor stemness, mechanistically linking mitochondrial Ca^2+^ homeostasis to PDAC chemoresistance.

Mitochondrial function is tightly regulated by Ca^2+^ signaling, which modulates ROS generation, metabolic plasticity, and electron transport chain activity to promote tumor progression [34, 35, 36]. Based on our clinical cohort, we found that patients with high MCU expression levels exhibit poorer responses to AG chemotherapy, and are associated with poor prognosis. As shown in Figure 3, genetic ablation of Mcu in autochthonous KPC mouse models delayed tumor initiation and reduced the expression of stemness markers, suggesting that MCU could promote PDAC progression and therapeutic resistance. Through the detection of Ca^2+^ signaling, we demonstrated that high expression of MCU in PDAC affects Ca^2+^ signaling between the ER and mitochondria via enhanced mitochondrial Ca^2+^ uptake. Both genetic knockout of MCU and MCUi4‐mediated pharmacological inhibition of mitochondrial Ca^2+^ influx were sufficient to significantly reverse tumor stemness and chemoresistance. Insufficient mitochondrial Ca^2+^ reduces oxidative phosphorylation (OXPHOS), whereas acute Ca^2+^ overload triggers mitochondrial dysfunction or apoptosis, particularly in research on cardiac diseases and neurodegenerative diseases. However, in PDAC, we observed that MCU expression levels gradually increase with disease progression (from pancreatic intraepithelial neoplasia (PanIN) to carcinoma) rather than being acutely activated. This increase occurs alongside tumor evolution and adaptive change, thereby protecting cells from chemotherapy‐induced apoptosis.

This adaptive change is reflected in metabolic reprogramming. Previous studies have shown that metabolites such as serine and lactate enhance chemoresistance in a variety of cancers through epigenetic modifications [20, 34]. As shown in Figure 4, multi‐omics analyses revealed significant activation of the *de novo* serine synthesis pathway and GSH synthesis pathway in MCU‐OE cells. GSH exists in reduced GSH and oxidized GSSG forms, serving as the primary ROS scavenger to protect stem‐like tumor cells from oxidative damage and enhances drug efflux [20, 37, 38]. Our findings indicate that MCU depletion sensitizes cancer stem cells to chemotherapy by disrupting redox balance. Supplementation with exogenous GSH or NAC restored spheroid formation capacity and therapeutic resistance in MCU‐KD cells. As shown in Figure 5, we identified PSAT1 and SLC7A11 as key MCU‐regulated metabolic genes. PSAT1, a critical enzyme in serine biosynthesis, facilitates epigenetic modifications, supports nucleotide synthesis [39], and maintains redox balance [27]. SLC7A11, which is upregulated in tumors under cystine‐deficient microenvironments, sustains GSH synthesis to prevent oxidative damage [40]; while its inhibition triggers substantial lipid peroxidation and ferroptosis [41]. Given that MCU deletion and serine/glycine deprivation exhibit significant synthetic lethality, we believe that MCU‐highly expressed tumors rely on metabolic reprogramming and redox homeostasis for survival.

We further explored how MCU‐mediated Ca^2+^ signaling governs this process. As shown in Figure 6, GO enrichment analysis of DEGs revealed that MCU triggers ER stress and UPR. We further identified that UPR‐mediated PERK phosphorylation may serve as a critical link in metabolic reprogramming [42, 43, 44]. As shown in Figure 7, PERK phosphorylation drives cellular adaptation to stress via ATF4 and NRF2, thereby contributing to tumor stemness. Mechanistically, Ca^2+^ influx activates the PERK‐NRF2 and PERK‐ATF4 signaling axes, which in turn orchestrate the transcriptional regulation of SLC7A11 and PSAT1. Notably, PERK inhibitors markedly abrogated this process even under conditions of active Ca^2+^ influx, suggesting the regulation of ATF4 and NRF2 by Ca^2+^ influx is dependent on UPR‐mediated PERK activation.

ER stress exerts bidirectional and dynamic control over cell fate: short‐term ER stress activation enhances GSH synthesis and amino acid metabolism, thereby protecting cells from oxidative damage. In contrast, sustained ER stress and accumulated oxidative damage upregulate pro‐apoptotic signaling cascades, shifting the balance toward cell death [45, 46]. Due to the accumulation of driver gene mutations, tumors tend to develop robust compensatory mechanisms to counteract stress. For instance, under ROS stress, certain TP53 mutations can promote survival under stress. The adaptive mechanisms in cancer cells with mutant TP53 include upregulation of the ROS scavenging system via NRF2 and activation of anti‐apoptotic signaling via NF‐κB [47]; KRAS mutations promote aspartate biosynthesis by upregulating ATF4 and PI3K‐AKT signaling to sustain tumor survival under oxidative stress [48, 49]. Given the high frequency of KRAS/TP53 co‐mutations in PDAC, MCU exerts precise regulation on ER stress, and through ATF4 and NRF2, ultimately enables PDAC to tolerate stress and survive.

High‐throughput screening of FDA‐approved drugs identified NB‐598 as a potent MCU inhibitor. Although we demonstrated that NB‐598 suppresses stemness by specifically targeting the RCOR1‐MCU axis, its mechanism and potential off‐target effects are complex and not fully elucidated. Future studies are therefore necessary to elucidate how NB‐598 modulates RCOR1‐mediated regulation of MCU. Additional experiments such as affinity chromatography and protein co‐crystallization should be employed to investigate whether NB‐598 exhibits higher binding affinity to uncharacterized off‐target proteins. Excitingly, the tumor‐suppressive effect of NB‐598 was highly comparable to that of MCUi4 (a well‐characterized MCU activity inhibitor), As shown in Figure 8, while NB‐598 exhibits reduced combined toxicity when administered with chemotherapy and extended the survival of mice with orthotopic tumor xenografts. Collectively, these observations suggest the potential advantages of NB‐598 for targeting PDAC patients with high MCU expression.

At the end of Figure 8, our proposed combination of NB‐598 and a serine/glycine‐free diet exacerbates the metabolic vulnerability of tumor cells and further enhances AG‐induced apoptosis. This is consistent with recent work by Tong et al. who reported that a serine/glycine‐free diet inhibit colorectal cancer cell growth and enhance antitumor immunity; their single‐arm phase I trial also demonstrated favorable feasibility and safety profiles. However, daily regimens exclusively composed of nutritional powders or low‐serine/glycine foods were associated with predominantly grade 1 or 2 gastrointestinal toxicity: diarrhea (65%), nausea (25%), and flatulence (20%) [50]. To address these issues, future protocols should consider implementing intermittent serine/glycine deprivation regimens or incorporating dietary fiber and probiotic supplementation into nutritional formulations. Notably, a serine/glycine‐free diet exerts a dual effects on antitumor immunity: it promotes tumor immune evasion via PD‐L1 lactylation while inducing ferroptosis in CD8^+^ T cells [50]. Given the high prevalence of cachexia in patients with advanced PDAC, a serine/glycine‐free diet may pose additional risks, including exacerbation of nutritional deterioration and potential induction of systemic multi‐organ complications. Thus, while a serine/glycine‐free diet represents a promising oncotherapeutic strategy, its clinical safety profile and potential adverse effects in patients require rigorous further investigation. Finally, our research has not addressed the impact of MCU expression on the tumor microenvironment. Since metabolic perturbations exert systemic effects, further investigations are needed to evaluate whether MCU enables tumor cells to co‐opts key stromal components (e.g., cancer‐associated fibroblasts, immune cells) to facilitate tumorigenesis and progression.

## Author Contributions

**Zekun Li**: conceptualization, investigation, writing – original draft, writing – review and editing; **Chenyang Meng**: investigation, resources; **Guangcong Shen**: methodology, visualization; **Yueying Shan**: data curation; **Junjin Wang**: investigation; **Diliyaer Abudukeremu**: formal analysis; **Rui Zhao**: formal analysis; **Bo Ni**: software; **Chao Xu**: software; **Xiaofan Guo**: writing – original draft; **Jun Yu**: writing – original draft; **Kaiyuan Wang**: writing – review and editing; **Yunzhan Li**: writing – review and editing; **Shengyu Yang**: validation; **Yongjie Xie**: project administration; **Tianxing Zhou**: funding acquisition; **Jihui Hao**: supervision; **Xiuchao Wang**: supervision, project administration, funding acquisition.

## Funding

The research was supported by grants from the Natural Science Foundation of China (grants 82273362 82472973); Tianjin Natural Science Foundation (22ICQNIC00100 24JCQNJC00390); The Science & Technology Development Fund of Tianjin Education Commission for Higher Education (2022K1221); Tianjin Health Research Project (Grant No.TJWJ2024QN016); The Science Technology Development Fund of the State Administration of Traditional Chinese medicine in Hebei Province (Grant No. T2025063); Tianjin Key Medical Discipline Construction Project (Grant No.TJYXZDXK‐3‐003A).

## Conflicts of Interest

The authors declare no conflicts of interest.

## Supporting information




**Supporting File 1**: advs73898‐sup‐0001‐SuppMat.docx.

**Supporting File 2**: advs73898‐sup‐0002‐TableS1‐S18.xlsx.

## Data Availability

The data that support the findings of this study are available on request from the corresponding author. The data are not publicly available due to privacy or ethical restrictions.
