# Supplementary material for: Mitochondrial Calcium Uniporter Drives Chemoresistance in Pancreatic Cancer via Glutathione‐Mediated Stemness Maintenance
Source: Adv Sci (Weinh). 2026 Jan 21;13(17):e07346. doi: 10.1002/advs.202507346 (PMC13042756; doi:10.1002/advs.202507346)
Supplement: Supplementary file 1 — Supporting File 1: advs73898‐sup‐0001‐SuppMat.docx. [file ADVS-13-e07346-s001.docx]

**Supplementary Methods**

**Validation Cohort**

The validation cohort data were downloaded from the GSA dataset (CRA001160). The data preprocessing pipeline was consistent with that of the primary analysis to ensure comparability. Standard quality control was performed using the Seurat R package. Cells were clustered into subpopulations via principal component analysis (PCA) and graph-based clustering methods. To replicate the primary analysis results, the identical stemness-associated gene signature was applied (see Supplementary Table 2 for details). The expression score of each epithelial cell in the validation dataset was calculated based on this gene signature. Subsequently, using the exact same scoring threshold as in the primary analysis, epithelial cells in the validation cohort were stratified into high-stemness and low-stemness epithelial cell clusters. To verify the expression pattern of the MCU gene, we compared the MCU expression levels between the aforementioned High-stemness and Low-stemness epithelial cell clusters. Differential expression analysis was performed using the Wilcoxon rank-sum test.

**Statistical analysis of single-cell RNA sequencing**

All statistical computations and visualizations were performed using R software (version 4.3.3) and relevant packages. Heatmaps were generated with the pheatmap package, while Venn diagrams for differential gene sets were created using the Venn package.

| **Table S1. Cox's proportional hazards model analysis of prognostic factors in patients with pancreatic cancer** | | | | | |
| --- | --- | --- | --- | --- | --- |
| **Overall survival** | | | | **Disease free survival** | |
| **Variables** | | **HR (95% CI)** | ***P* values** | **HR (95% CI)** | ***P* values** |
| **Univariate analysis** | | | | | |
| **Sex** | Female / Male | 0.894 (0.588-1.358) | 0.599 | 0.775 (0.521-1.153) | 0.209 |
| **Age** | ≥65 /＜65 | 1.174 (0.768-1.795) | 0.460 | 1.081 (0.729-1.603) | 0.698 |
| **Tumor size** | ≥3.5 cm /＜3.5 cm | 1.388 (0.867-2.222) | 0.172 | 1.147 (0.710-1.853) | 0.576 |
| **Differentiation** | Well / moderate or poor | 4.205 (2.332-7.583) | **< 0.001*** | 3.357 (2.009-5.611) | **< 0.001*** |
| **TNM** | Ⅰ and ⅠⅠA / ⅠⅠB and III | 1.635 (1.171-2.495) | **0.002*** | 1.710 (1.149-2.544) | **0.003*** |
| **MCU** | Low (– / +) / High (++ / +++) | 1.872 (1.235-2.836) | **0.000*** | 1.721 (1.153-2.568) | **0.004*** |
| **Multivariateanalysis** | | | | | |
| **Differentiation** | Well / moderate or poor | 3.746 (2.271-6.181) | **< 0.001*** | 2.990 (1.944-4.598) | **< 0.001*** |
| **TNM** | Ⅰ and ⅠⅠA / ⅠⅠB and III | 1.522 (1.024-2.215) | **0.003*** | 1.646 (1.181-2.363) | **0.008*** |
| **MCU** | Low (– / +) / High (++ / +++) | 1.758 (1.198-2.690) | **< 0.001*** | 1.586 (1.098-2.314) | **0.006*** |
|  | |  |  |  |  |
| * *P* < 0.05; HR, hazard ratio; CI, confidence interval. | | | | | |
| Univariate analysis: log rank; Multivariate Cox's analysis method: Forward: LR | | | | | |

**Immunohistochemistry (IHC)**

IHC was used to analyze the expression of MCU in PDAC tissue samples. Paraffin-embedded PDAC specimens on slides were sequentially treated with xylene, absolute ethanol, 95% ethanol, 85% ethanol, and 75% ethanol to remove surface paraffin, followed by high-temperature heating for 5 minutes to retrieve the antigen. Sequential sections were used to evaluate the co-expression of proteins including MCU, MICU1, MICU2, MICU3, PSAT1, and SLC7A11. Then, the slides were incubated with the primary antibody at 4°C overnight. Next, the sections were incubated with the secondary antibody at 37°C for 30 minutes. The DAB Substrate Kit (Vazyme, Cat: HC301-02) was used for color development. The staining intensity was classified as follows: 1 (low) and 3 (high). The staining extent was categorized as: 0–50% and 51–100%. Five random fields were examined under an optical microscope at 100× magnification. IHC scoring was determined by two independent pathologists who were blinded to the clinical characteristics and outcomes of the patients. Details of the primary antibodies used can be found in Supplementary Table 11.

**Multiplex immunofluorescence staining and imaging**

Tumor tissue sections (5 μm thick) were deparaffinized with xylene (2 × 30 minutes) and rehydrated through a graded ethanol series (100% ethanol, 2 × 20 minutes; 95% ethanol, 1 × 10 minutes; 85% ethanol, 1 × 10 minutes; followed by rinsing in 75% ethanol). Antigen retrieval was conducted using a microwave-heated EDTA buffer for 15 minutes with the EZ Retriever Microwave System. To minimize non-specific binding, protein blocking was performed using blocking buffer (Akoya) before incubation with primary antibodies and horseradish peroxidase (HRP)-conjugated polymer secondary antibodies (Akoya). Next, a second round of antigen retrieval was carried out in heated EDTA buffer for 15 minutes to remove unbound antibodies, ensuring the tissue was prepared for subsequent staining. Nuclei were counterstained with DAPI for 10 minutes, and slides were mounted using Fluorescence Mounting Medium (Thermo Fisher Scientific) in a phosphate-free process. Stained slides were scanned using the Tissue-Gnostics System. Regions of interest were systematically selected using an unbiased grid-based approach to ensure tissue heterogeneity was captured. Images were acquired with a ×20 objective, and unstained tissue sections were used to extract autofluorescence. Image analysis was performed using TissueGnostics analysis software 2.4. Details of the primary antibodies used can be found in Supplementary Table 11.

**RNA extraction and qPCR**

Total RNA was isolated from the specified cells using TRIzol reagent (Invitrogen) and subsequently reverse transcribed into cDNA using the StarScript III All-in-One RT Mix with gDNA Remover Kit according to the manufacturer’s instructions. qPCR was conducted to assess target gene expression at the cDNA level, with each experiment repeated independently at least three times. Actin served as the internal control. RNase-free conditions were strictly maintained throughout the entire procedure to prevent contamination. Details of the primers used can be found in Supplementary Table 12.

**Chromatin immunoprecipitation (ChIP) and dual-luciferase reporter assay**

ChIP assays were performed using a ChIP Kit (Millipore) according to the manufacturer’s instructions. Briefly, cells were immunoprecipitated with anti-ATF4 or anti-NRF2 antibodies. The immunoprecipitated products were detected by PCR. Related sequences are listed in Supplementary Table 7. Luciferase analysis was performed on the basis of the binding sites identified through ChIP analysis. PDX-4 cells transfected with pCDH-MCU or pCDH-vector were transfected with the following vectors: pGL3-SLC7A11-BS-wt, pGL3-SLC7A11-BS1-mutation, pGL3-SLC7A11-BS2-mutation, pGL3-SLC7A11-BS1+2-mutation, pGL3-PSAT1-BS-mutation, pGL3-PSAT1-BS1-mutation, pGL3-PSAT1-BS2-mutation, pGL3-SLC7A11-BS1+2-mutation and pGL3-empty vectors (EV). After 48 hours, cells were subjected to dual-luciferase reporter analyses. Details of the primers used can be found in Supplementary Table 13.

**Organoid construction**

Organoids derived from PDAC specimens were isolated and cultured following established methods. Specifically, fresh PDAC tissue was cut into small pieces (<1 mm³) and washed three times with cold PBS buffer containing 10% penicillin and streptomycin. The tissue was then incubated in a digestion solution containing 1% fetal bovine serum (FBS), 10% penicillin and streptomycin, 1.5 mg/mL Type II collagenase, 500 U/mL Type IV collagenase, 0.1 mg/mL Type II dispase, and 10 mM Y-27,632 (Selleck, Shanghai, China) at 37°C with gentle shaking for 45 minutes. After digestion, tumor fragments were washed three times with cold PBS and collected by centrifugation at 500 g for 5 minutes. The tumor cells were then embedded in Matrigel (BD Bioscience, Shanghai) and seeded into 96-well plates. After Matrigel solidification, the organoids were cultured in complete Advanced DMEM/F12 medium (Thermo Fisher Scientific), supplemented with Noggin (0.1 mg/mL, Peprotech), R-spondin (1 μg/mL, Nuvelo), epidermal growth factor (EGF, 50 ng/mL, Peprotech), Glutamax (Invitrogen), HEPES (Invitrogen), N2 (Invitrogen), B27 (Invitrogen), N-acetyl-L-cysteine (1 mM, Sigma-Aldrich), gastrin (10 nM, Sigma-Aldrich), nicotinamide (10 mM, Sigma-Aldrich), A83-01 (0.5 mM, Tocris Bioscience), and fibroblast growth factor 10 (FGF10, 100 ng/mL, Peprotech). The organoid culture medium was replaced approximately every 3 days, and organoids were passaged every 7 days based on their growth status.

**Drug synergy analysis**

Synergy scores were calculated using SynergyFinder version 2.0 (available at <https://synergyfinder.fimm.fi>). Interpretation of the final synergy scores was as follows: a score below -10 indicates an antagonistic interaction between the two drugs; scores between -10 and 10 reflect an additive effect; and scores above 10 suggest a synergistic interaction.

**Liquid chromatography with tandem mass spectrometry (LC-MS/MS) analysis of GSH**

Cells (2 × 10^5^ cells/well) were seeded into 12-well plates and cultured until they reached 70% confluency. Then, they were washed three times with ice cold 1 × PBS. Residual PBS was thoroughly removed. Ice-cold 0.1% formic acid (225 μL) and [^13^C_2_,^15^N] GSH (25 μL; 1 μg/mL in 0.1% formic acid) were added to each well. Cells were scraped, placed in a 1.5 mL Eppendorf tube, vortexed for 15 seconds, snap-frozen in liquid nitrogen for 1 minute and thawed in a room temperature water bath. After three freeze-thaw cycles, cells were centrifuged at 13,000 g for 3 minutes and the supernatant was collected for LC-MS/MS analysis.

**^13^C_6_-glucose tracing**

For glucose tracing, control scramble or MCU-KD cells (5 × 10^6^) were incubated in DMEM containing 10 mM ^13^C_6_-glucose and 10% dialyzed FBS for the indicated time (from 0–360 minutes). The supernatant was collected and dried using SpeedVac and re-dissolved in 50 μL of methanol/water (50/50) and analyzed by LC-MS/MS.

**^13^C_6_,^15^N_2_-cystine tracing**

For cystine isotopic tracing, cystine-, methionine- and glutamine-free DMEM (Gibco, Cat. No.: 21013-024) was supplemented with 50 μM ^13^C, ^15^N-cysteine**,** 100 μM methionine, 4 mM glutamine and 10% dialyzed FBS and stored at 4°C for 48 hours for the oxidation of cysteine to cystine. Control scramble or MCU-KD cells (5 × 10^6^) were pre-conditioned in the same medium containing ^12^C,^14^N-cystine for 12 hours. After pre-conditioning, cells were labeled with their respective media containing isotopic cystine for the indicated time (2, 4 or 24 hours). Metabolites were extracted with 2 mL 80% MeOH. After centrifugation, the supernatant was dried under nitrogen flow and analyzed by LC-MS/MS.

**Generation of the KPMC mouse model**

*Mcu^loxp/loxp^*; *Pdx-1-Cre* mice were purchased from Shanghai Model Organisms and subsequently crossed with *Kras^G12D/+^*; *Trp53^loxp/loxp^* mice, which were bred in our laboratory, to generate the KPMC model. Tumor development and progression were monitored using ultrasound imaging (**VisualSonics Vevo 2100, Canada**). Mice with tumor volumes ranging from **20 to 60 mm³** were randomly assigned to each treatment group. Details of the primers used and PCR procedure can be found in Supplementary Table 14-15.

***In vivo* extreme limiting dilution tumorigenesis assay**

Tumor cell suspension was mixed with Matrigel at a 2 : 1 ratio to a final volume of 50 μL. Tumor cells were then implanted subcutaneously into 4-5 week-old female BALB/c mice at three different concentrations: 1 × 10⁶, 1 × 10⁵, and 1 × 10³ cells per mouse, with 6 or 7 mice per group. The tumor size was measured regularly, and tumor formation rates were recorded after 3 weeks. Stem cell frequencies were statistically analyzed using ELDA (Extreme Limiting Dilution Analysis).

**Detection of intracellular Ca²⁺ signals**

Mitochondrial Ca^2+^ level was assessed using a genetically encoded Ca^2+^ indicator. Cells were transfected with the mitochondrial-targeted Ca^2+^ sensor pLX304-CEPIA3mt. After transfection, cells underwent selection to enrich for positive transfection, which were subsequently verified by fluorescence microscopy. Prior to imaging, transfected cells were pretreated with indicated compound for 24 hours. On the day of imaging, cells were washed and maintained in 1 x Hank’s Balanced Salt Solution (HBSS, Gibco, 14025092) at 37°C. Live-cell imaging was performed on a Leica SP8 confocal microscope, with time-lapse images acquired every 2 seconds for a total duration of 15 minutes. To stimulate mitochondrial Ca^2+^ uptake, 2.5 μM ionomycin (Iono) was added directly to the imaging buffer during acquisition, followed by the addition of 1 mM CaCl₂. For the detection of cytosolic and ER Ca^2+^ level, cells were transfected with the Ca^2+^ probe GCaMP6f and G-CEPIA1er, 2 μM thapsigargin (Tg) was used to induce ER Ca^2+^ release. Regions of interest (ROIs) were manually drawn around individual cells, and the mean fluorescence intensity over time was extracted for each ROI. Fluorescence intensity data were collected from sixty ROIs, derived from three independent plates per experimental group and the mean fluorescent intensity (MFI) of the corresponding regions was exported for analysis.

**Cell counting kit-8 (CCK-8) assay**

Cells were plated in 96-well plates at a density of approximately 5 × 10³ cells per well. Once the cells reached 80–90% confluence, the culture medium was replaced with 100 μL culture medium containing 10% CCK-8 reagent and cells were incubated at 37°C in the dark for 2 hours. Absorbance was measured using a dual-wavelength detection method at a wavelength of 450 nm with a reference wavelength of 600 nm.

**TUNEL staining assay**

Formalin-fixed tumor tissues were embedded in paraffin and sectioned at a thickness of 5 μm. Following the manufacturer’s instructions (Roche), the samples were incubated with TUNEL mix, followed by treatment with 5% TUNEL enzyme and TUNEL label (Roche). DNase I (50 U/μl, Sigma-Aldrich)-treated samples served as positive controls, while negative control samples were incubated with TUNEL label only. Apoptotic cells were identified by localized green fluorescence, and DAPI staining was used to visualize cell nuclei. Images were acquired using a Zeiss fluorescence microscope at 400× magnification.

**Metabolite Extraction and NADPH Quantification by LC-MS/MS**

Cells were seeded at 1 × 10⁶ cells/well in 6-well plates 1 day prior. Medium was fully replaced 2 h before metabolite collection. For extraction: medium was aspirated completely, wells were washed twice with 2 mL room-temperature PBS, then 0.4 mL pre-chilled (-20°C) 80% methanol (20% double-distilled water, DDW) was added. Plates were immediately placed on dry ice, incubated at -80°C for 15 min, then contents were scraped into 1.5 mL tubes. Samples were centrifuged at 15,000× g for 10 min at 4°C, and supernatants were collected. NADPH standards (1 mg/mL stock in water) were serially diluted from 10,000 ng/mL to 1–500 ng/mL in 80% methanol (5 μL standard + 45 μL 80% methanol). For LC-MS/MS: Harvested cells were PBS-washed, extracted with 80% methanol, vortexed, and centrifuged at 21,130 ×g (4°C, 10 min). Supernatants were analyzed on a Sciex QTRAP 6500+ coupled with EXion HPLC, using a 3 μm Sequant ZIC-cHILIC column (2.1 × 150 mm). Mobile phases A (10 mM NH₄Ac in 90% H₂O/10% acetonitrile) and B (5 mM NH₄Ac in 10% H₂O/90% acetonitrile) were run at 0.4 mL/min with gradient: 10% A/90% B → 34% B over 2.5 min (hold 1.5 min) → 55% A over 0.5 min, then re-equilibration. Mass spectrometry (positive ESI, MRM) used parameters: CUR 35 L/h, CAD medium, ion spray 5500 V, 400°C, gas 1/2 at 40/30 L/h. NADPH: DP 75 V, EP 10 V, CE 29.4 V, CXP 18 V; transition m/z 746→729. Quantification was via Sciex OS 3.0 software.

**Cellular Thermal Shift Assay (CETSA)**

Cells were seeded in 10 cm dishes to reach 70–80% confluency then treated with 5 μM NB-598 or vehicle control for 24h. Then cells were washed with ice-cold PBS, collected in 1 mL PBS with protease inhibitors to form a single-cell suspension, and aliquot 100 μL into 10 PCR tubes. Incubate each tube at designated temperatures (37, 41, 45, 49, 53, 57, 61, 65, 69 °C) for 3 minutes, freeze in liquid nitrogen for 3 minutes, and repeat for 3 total freeze-thaw cycles. Centrifuge lysates at 20,000 ×g for 20 minutes at 4°C, collect supernatants, quantify protein, prepare samples, and perform Western blot to detect target protein and assess thermal stability shifts.

**Detection of ROS**

Intracellular ROS levels were quantified using the ROS Assay Kit (TEASEN, Cat. No.: 50101ES01). Cells were incubated with 5 μM DCFH-DA in pre-warmed HBSS at 37°C for 30 minutes. After staining, cells were washed twice with PBS and resuspended in PBS. Fluorescence signals were detected using a flow cytometer with an excitation wavelength set at 488 nm and an emission wavelength set at 525 nm.

**Integrated Transcriptomic and Metabolomic Analysis**

PDX4-Vector and PDX4-MCU-OE cells were seeded separately into 10 cm dishes. At ~80% confluency, cells were harvested and split equally into two aliquots for metabolomic and transcriptomic analyses (n = 3 biological replicates/group). For transcriptomic sequencing: Total RNA was extracted using the Qiagen RNeasy Kit (Qiagen), and sequencing libraries were constructed with the NEB Next Ultra RNA Library Prep Kit for Illumina platform sequencing. After filtering low-expression genes, gene expression levels were quantified as CPM, and differentially expressed genes (DEGs) were identified via DESeq2 with thresholds of |log₂ (FC)| ≥ 1 and adjusted *P*-value < 0.05. Detailed gene lists are provided in Supplementary Table 16. For metabolomic sequencing: Cells were snap-frozen in liquid nitrogen prior to metabolite extraction and analyzed by UPLC-MS/MS. Metabolite Set Enrichment Analysis (MSEA) was performed using MetaboAnalyst 5.0 and metabolite sets with a false discovery rate (FDR) < 0.25 were considered significantly enriched. Detailed metabolite lists are provided in Supplementary Table 17.

For multi-omics data integration, datasets were first standardized: synonymous annotations were unified in accordance with the Kyoto Encyclopedia of Genes and Genomes (KEGG) pathway naming conventions, and redundant spaces in pathway labels were removed. Subsequently, KEGG-enriched pathways were retrieved from DEG enrichment analysis (transcriptome) and MSEA (metabolome). Intersection analysis of these two pathway sets identified 20 overlapping pathways, designated as "co-enriched pathways". These co-enriched pathways are summarized in a structured table, including pathway names and corresponding adjusted *P*-values. To visually illustrate the results of the integrated analysis, a grouped bar chart was generated. Raw data and statistical parameters from the joint pathway screening are provided in Supplementary Table 18.

**Supplementary Figures**

**
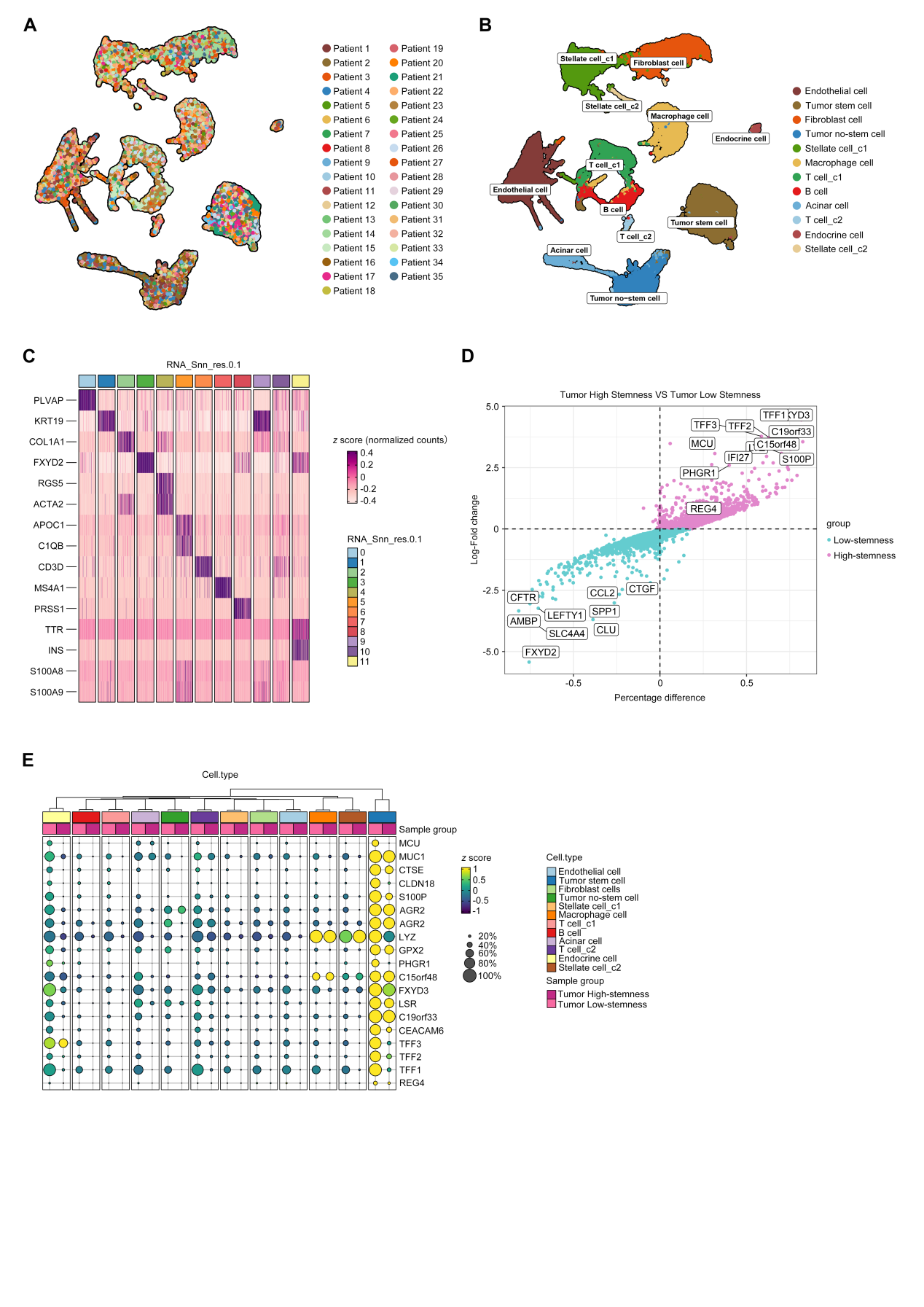
Supplementary Figure S1. Cross-dataset validation reveals MCU upregulation in high-stemness PDAC ductal clusters.**

(A) UMAP plot showing cell clustering analysis of specimens from 35 PDAC patients. (B) UMAP plot revealing 12 distinct populations of cells. (C) Heatmap showing the relative expression levels of specific marker genes in each cell population as z-score values. Expression levels range from white (low) to purple (high). (D) Scatter plot showing differentially expressed genes between high-stemness and low-stemness tumor groups. (E) Bubble plot showing the expression levels of indicated genes across different cell subpopulations and ductal cell clusters grouped by high-stemness and low-stemness.

**
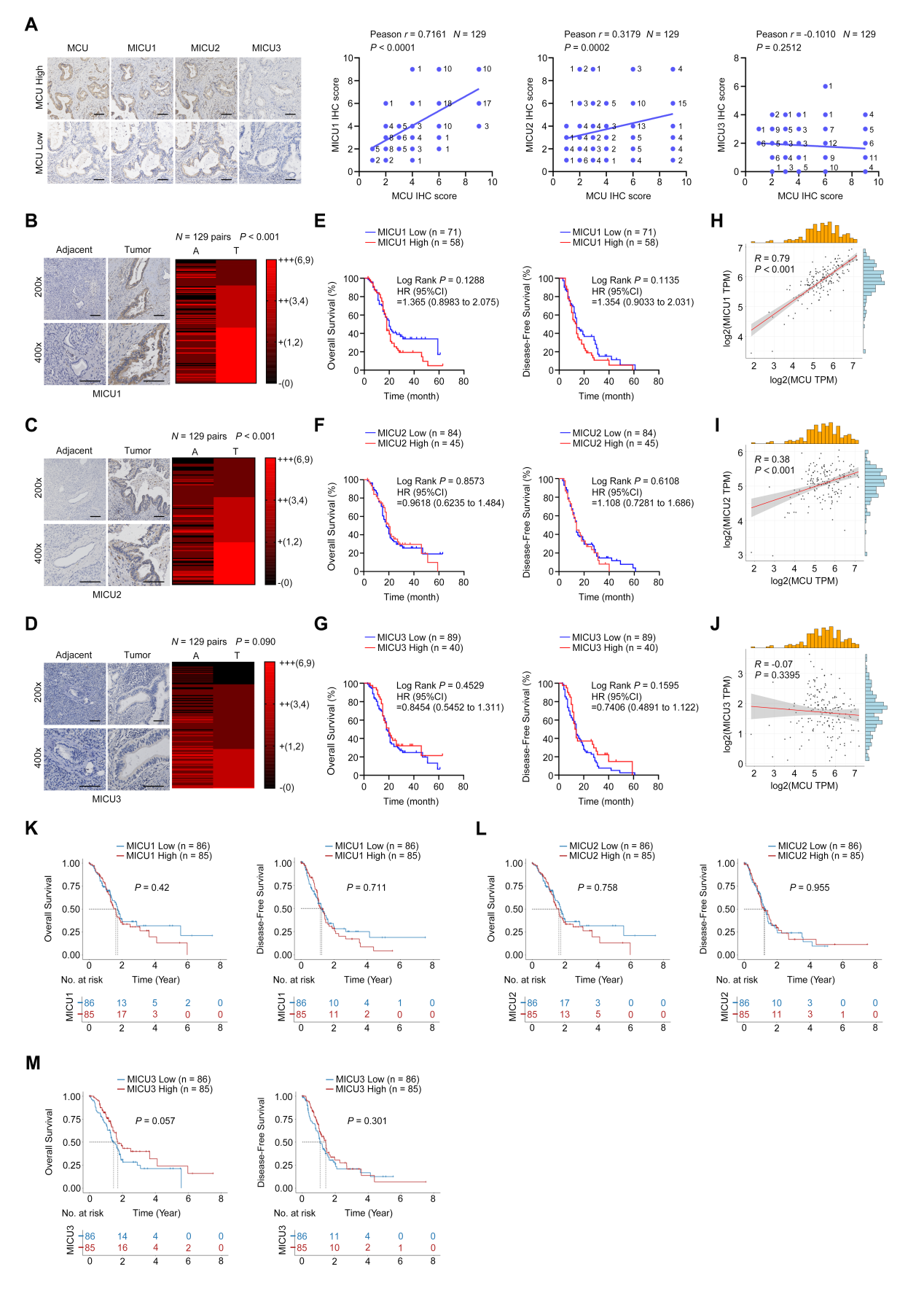
Supplementary Figure S2. Correlations of regulatory subunits with MCU and their prognostic significance in pancreatic cancer**

1. MCU, MICU1, MICU2, and MICU3 IHC staining in sections of 129 PDAC patients and Pearson correlation between MCU and MICU1, MICU2, and MICU3 protein expression levels are shown. Sequential sections were used for the IHC staining. (B-G) The association between MICU1, MICU2, and MICU3 expression levels and patient prognosis. Representative IHC images of the MICU1 (B), MICU2 (C), and MICU3 (D) protein level in 129 PDAC tissues and paired adjacent non-tumor tissues. Scale bar, 100 µm. The expression level is categorized as absent– (0), weak+ (1,2), moderate++ (3,4), and strong+++ (6,9). Heatmap showing a comparison of these protein levels between PDAC tissues (T) and paired adjacent non-tumor tissues (A). Kaplan–Meier curves showing OS or DFS in a cohort of 129 PDAC patients stratified by MICU1 (E), MICU2 (F), and MICU3 (G) protein levels. Hazard Ratios (HR) with 95% Confidence intervals (CI) are calculated via the Cox proportional hazards model; log-rank test *p-*values are indicated. (H-M) The TCGA database was used to validate the results of A-G. Scatter plot showing the correlation between MCU and MICU1 (H), MICU2 (I), and MICU3 (J) mRNA expression levels. Kaplan–Meier curves showing the correlation between MICU1 (K), MICU2 (L), and MICU3 (M) mRNA expression levels and OS or DFS in a cohort of 171 PDAC patients from the TCGA database. Data in (B-D) were analyzed using Wilcoxon signed-rank test. Data in (E-G) and (K-M) were analyzed using two-tailed log-rank test.


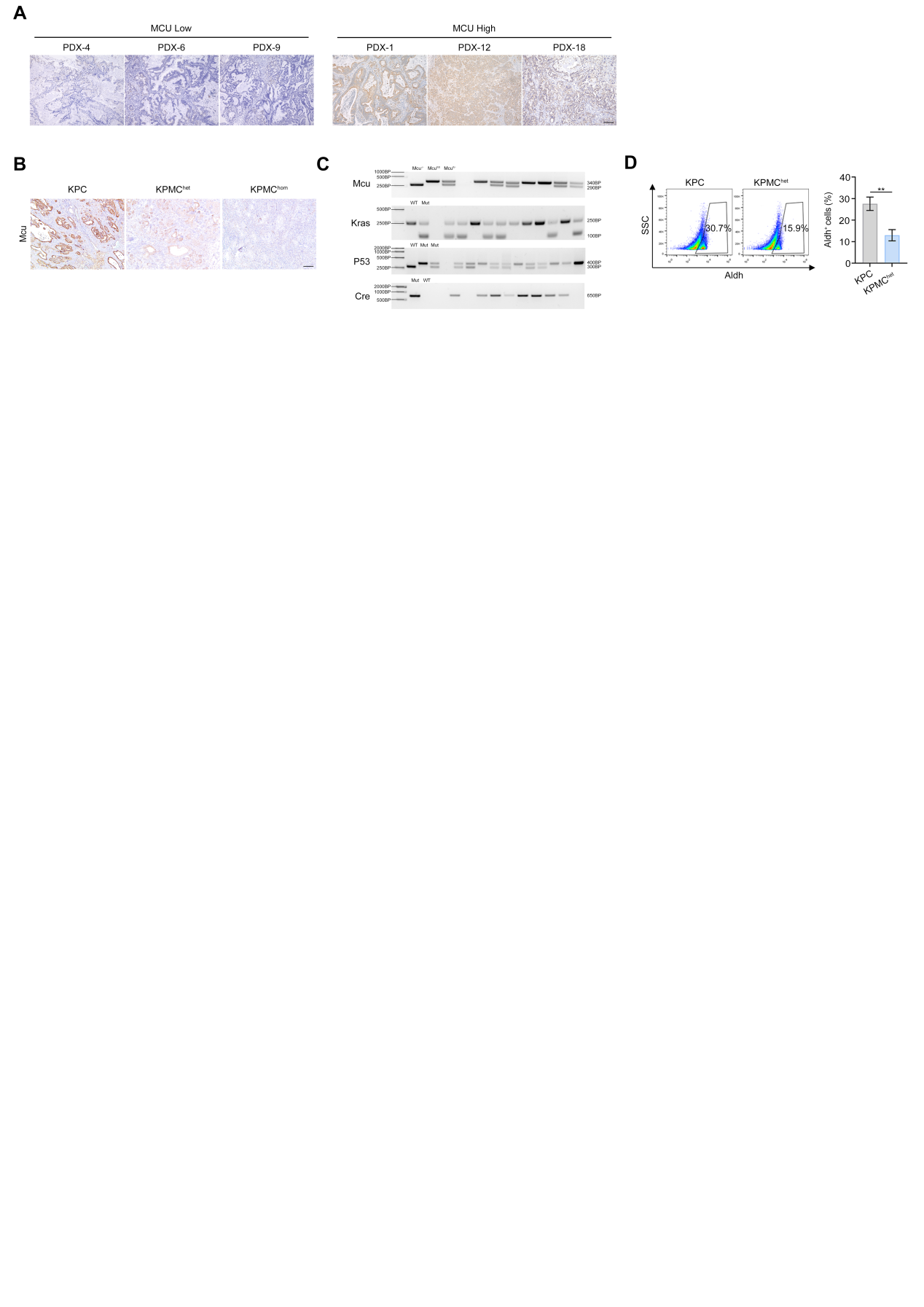


**Supplementary Figure S3. Evaluation of MCU in patient specimens and a genetically engineered mouse model.**

(A) Representative images showing MCU IHC staining in the indicated PDXs. (B) Representative images showing Mcu IHC staining in KPC (Mcu Wild-type), KPMC^het^ (Mcu Heterozygous Knockout) and KPMC^hom^ (Mcu Homozygous Knockout) tumors. (C) PCR agarose gel assays were used to identify the genotype of KPC, KPMC^het^ and KPMC^hom^ mice. (D) Flow cytometry was used to determine the proportion of Aldh^+^ cells within the epithelial cell population isolated from KPC and KPMC^het^ mice. Data in (D) are presented as mean ± SD of 3 biological replicates and were analyzed using two-sample, two-tailed unpaired Student’s *t*-test. ns, not significant, **P* < 0.05, ***P* < 0.01, ****P* < 0.001 and *****P* < 0.0001. Scale bar, 100 μm.

**
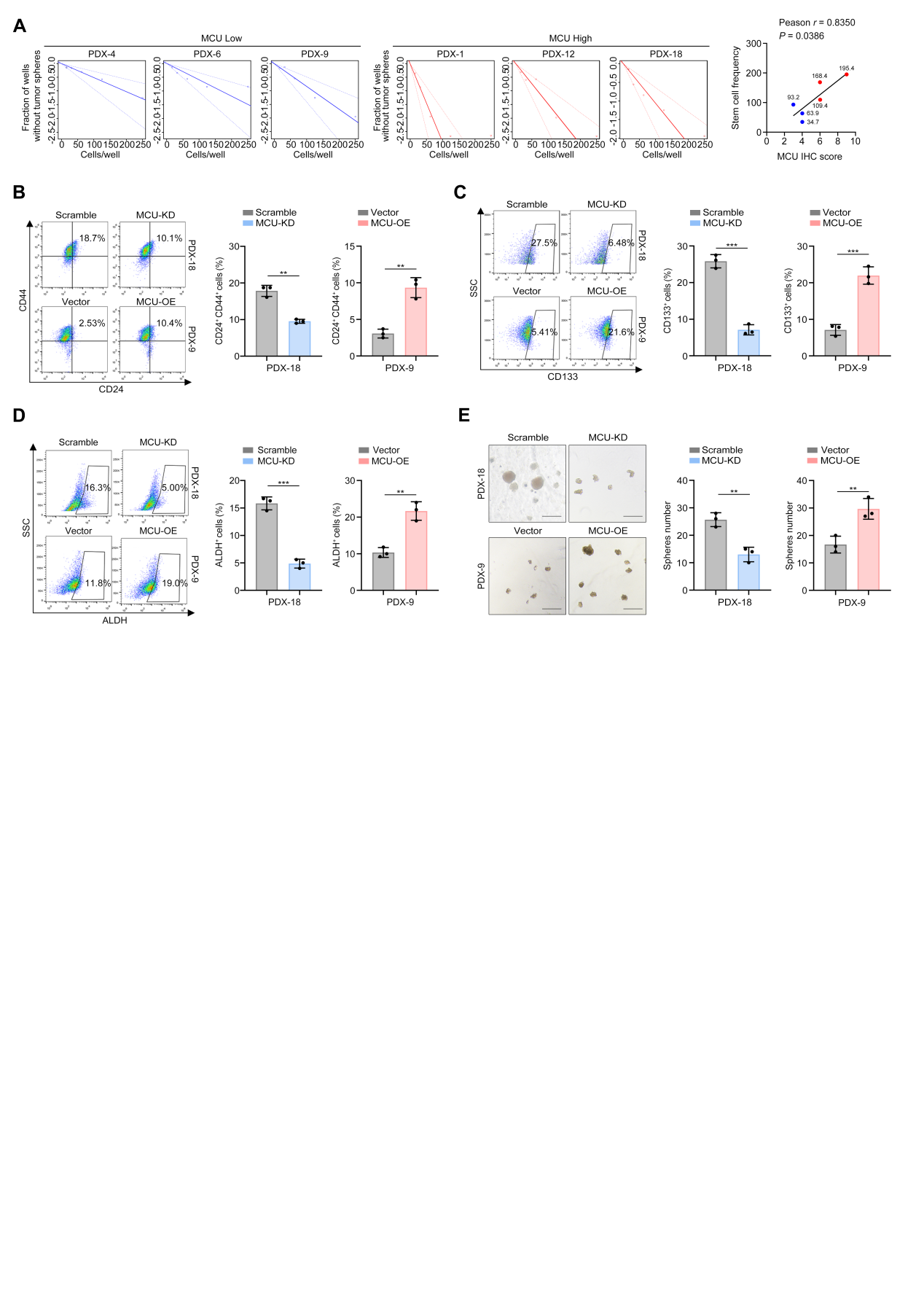
**

**Supplementary Figure S4. MCU is responsible for stemness maintenance.**

1. *In vitro* limited dilution assays were performed in a panel of PDX cell lines with different MCU protein expression levels. Pearson correlation analysis showing the correlation between the MCU expression level and stem cell frequency. (B-E) Flow cytometry was used to determine the proportions of CD44^+^ CD24^+^, CD133^+^ and ALDH^+^ cells in the indicated cells. Representative dot plots and percentage of CD44^+^ CD24^+^ cells (B), CD133^+^ cells (C) and ALDH^+^ cells (D) are shown. (E) Sphere formation assays were performed in the indicated cell lines. Representative images and quantification of sphere numbers are shown. Scale bar, 200 µm. Data in (B-E) are presented as mean ± SD of 3 biological replicates and were analyzed using two-sample, two-tailed unpaired Student’s *t*-test. ns, not significant, **P* < 0.05, ***P* < 0.01, ****P* < 0.001 and *****P* < 0.0001.

**
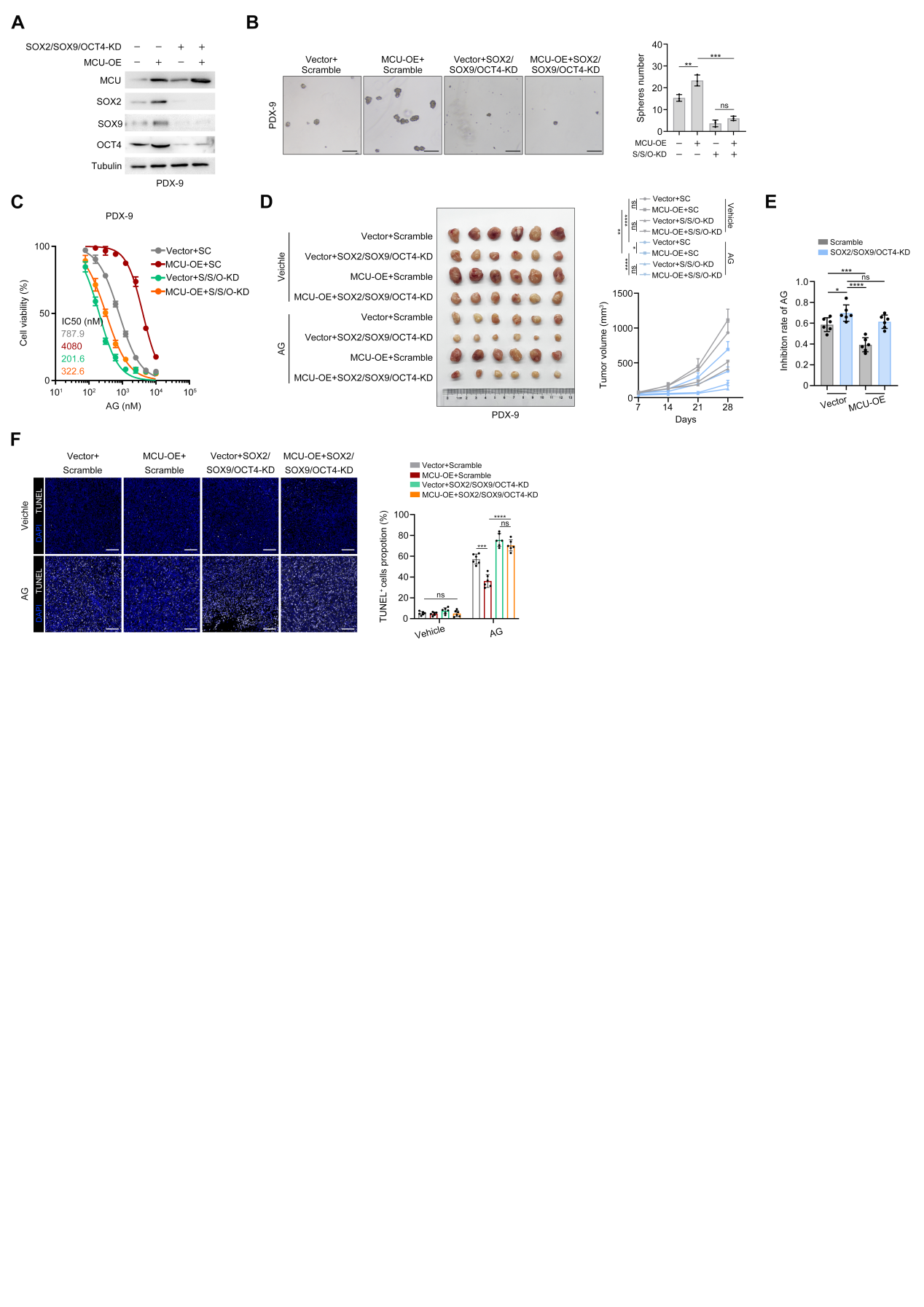
Supplementary Figure S5. MCU mediates chemoresistance by promoting stemness maintenance.**

1. Western blots showing the knockdown efficiency of SOX2, SOX9, and OCT4 in PDX-9-Vector and PDX-9-MCU-OE cells. (B) The effects of SOX2, SOX9, and OCT4 triple knockdown on sphere formation capacity in PDX-9-MCU-OE and PDX-9-Vector control cells. Scale bar, 100 µm. (C) Dose response curves showing the effects of SOX2, SOX9, and OCT4 triple knockdown on sensitivities to AG treatment in PDX-9-MCU-OE and PDX-9-Vector control cells. (D-F) The effects of SOX2, SOX9, and OCT4 triple on chemoresistance *in vitro*. Immunocompromised BALB/c nude mice were subcutaneously inoculated with the indicated cells (n = 6 per group). Mice were administered vehicle or AG 7 days later (defined as Day 0) and humanely killed on Day 28. Representative tumor images and growth curves (D), inhibition rates (E) and IHC staining with the quantification of TUNEL^+^ cells (F) are shown. Data in (B-C) showed mean±SD from 3 biological replicates. Data in (D) were analyzed using two-way ANOVA followed by Tukey’s multiple comparison test. Data in (B), and (E-F) are presented as mean ± SD and were analyzed using two-sample, two-tailed unpaired Student’s *t*-test. ns, not significant, **P* < 0.05, ***P* < 0.01, ****P* < 0.001 and *****P* < 0.0001.

**
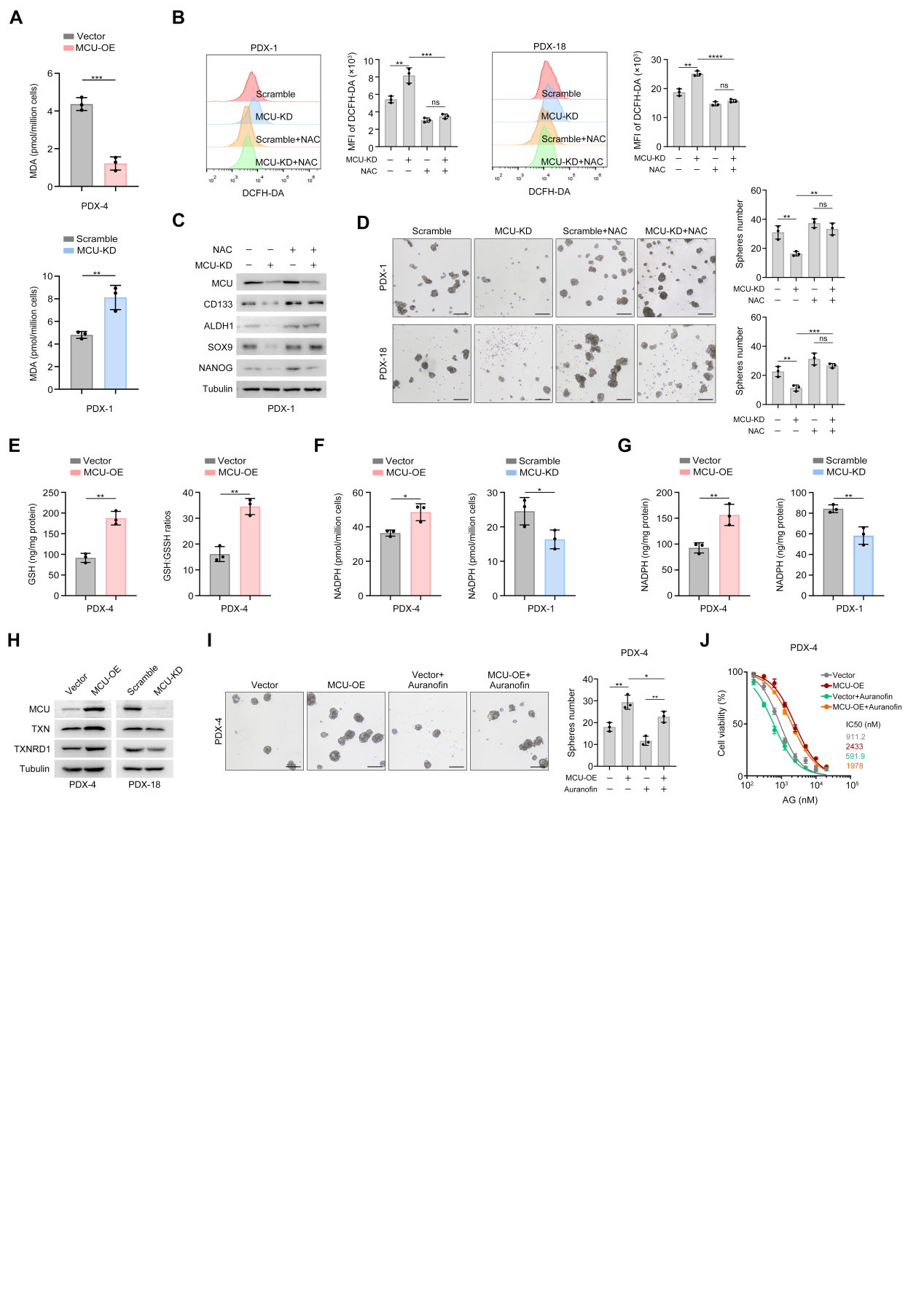
**

**Supplementary Figure S6. MCU maintains stemness through GSH-mediated ROS scavenging.**

(A) The effects of MCU overexpression and MCU knockdown on intracellular MDA levels. (B-D) The effects of ROS scavenging on MCU-mediated stemness maintenance. Cells were subjected to NAC supplementation, then the detections of intracellular ROS levels (B), stemness marker expression (C), and sphere formation capacity (D) in MCU-KD and Scramble control PDX cells are shown. (E) The effects of MCU knockdown on intracellular GSH levels and GSH:GSSG ratios in PDX-4 cells. (F-G) The effects of MCU overexpression and MCU knockdown on intracellular NADPH level. Cells were measure by ELISA (F) and LC-MS (G). (H) Western blots showing the effects of MCU overexpression and MCU knockdown on cellular TXN and TXNRD1 expression. (I) The effects of thioredoxin system inhibitor on sphere formation capacity in MCU-OE and Vector control PDX-4 cells. Scale bar, 100 µm. (J) Dose response curves showing the effects of thioredoxin system inhibitor on sensitivities to AG treatment in MCU-OE and Vector control PDX-4 cells. Data in (A-B), (D-G), and (I-J) are presented as mean ± SD of 3 biological replicates and were analyzed using two-sample, two-tailed unpaired Student’s *t*-test. ns, not significant, **P* < 0.05, ***P* < 0.01, ****P* < 0.001 and *****P* < 0.0001.


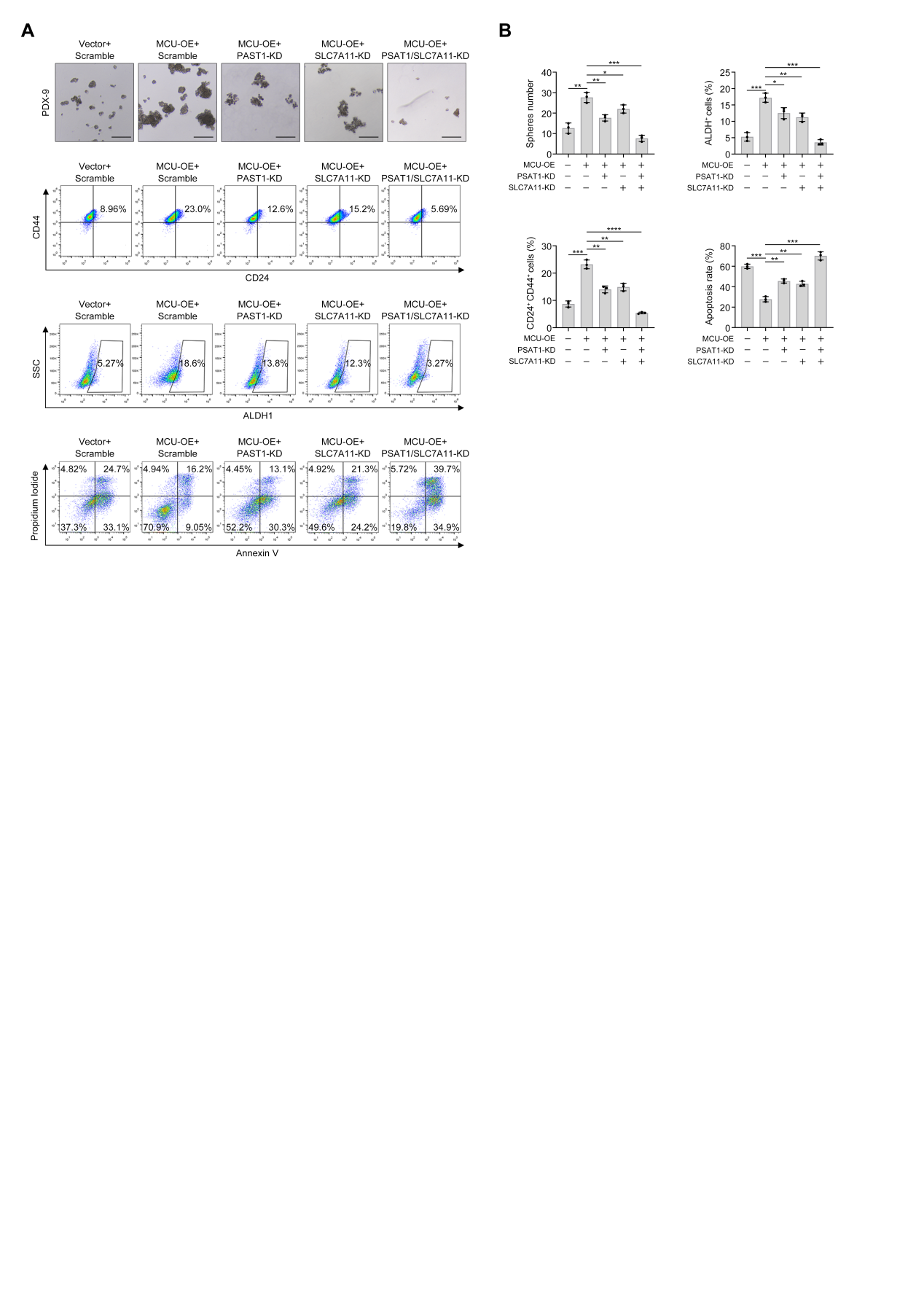


**Supplementary Figure S7. Knockdown of PSAT1 and SLC7A11 restores MCU-mediated PDAC stemness and chemoresistance.**

(A-B) The effects of PSAT1 knockdown and SLC7A11 knockdown on MCU-mediated stemness maintenance and chemoresistance *in vitro*. Representative images (A) and quantifications (B) of sphere formation capacity, CD24^+^ CD44^+^ proportion, ALDH^+^ proportion and apoptotic rate following AG treatment of PDX-9 cells in the indicated groups are shown. Scale bar, 100 µm. Data in (A-B) are presented as mean ± SD of 3 biological replicates and were analyzed using two-sample, two-tailed unpaired Student’s *t*-test. ns, not significant, **P* < 0.05, ***P* < 0.01, ****P* < 0.001 and *****P* < 0.0001.

**
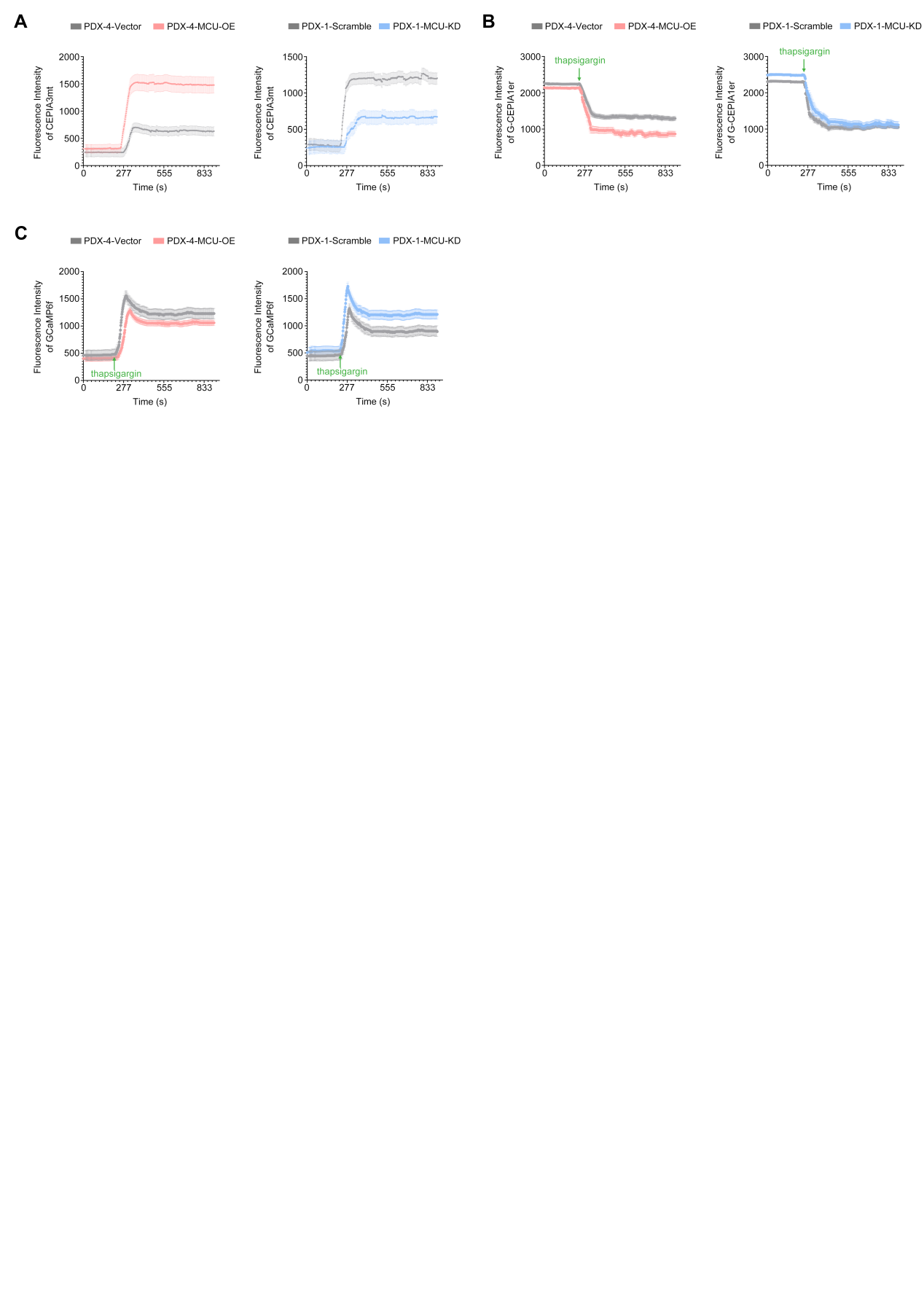
**

**Supplementary Figure S8.** **The expression of MCU affects Ca^2+^ influx from the ER to mitochondria in PDAC.**

1. The effects of MCU overexpression (left) and MCU knockdown (right) on mitochondrial Ca^2+^ uptake in PDX cells. (B) The effects of MCU overexpression (left) and MCU knockdown (right) on ER Ca^2+^ release in PDX cells. (C) The effects of MCU overexpression (left) and MCU knockdown (right) on cytosolic Ca²⁺ influx in PDX cells. All experiments were repeated three times independently.

**
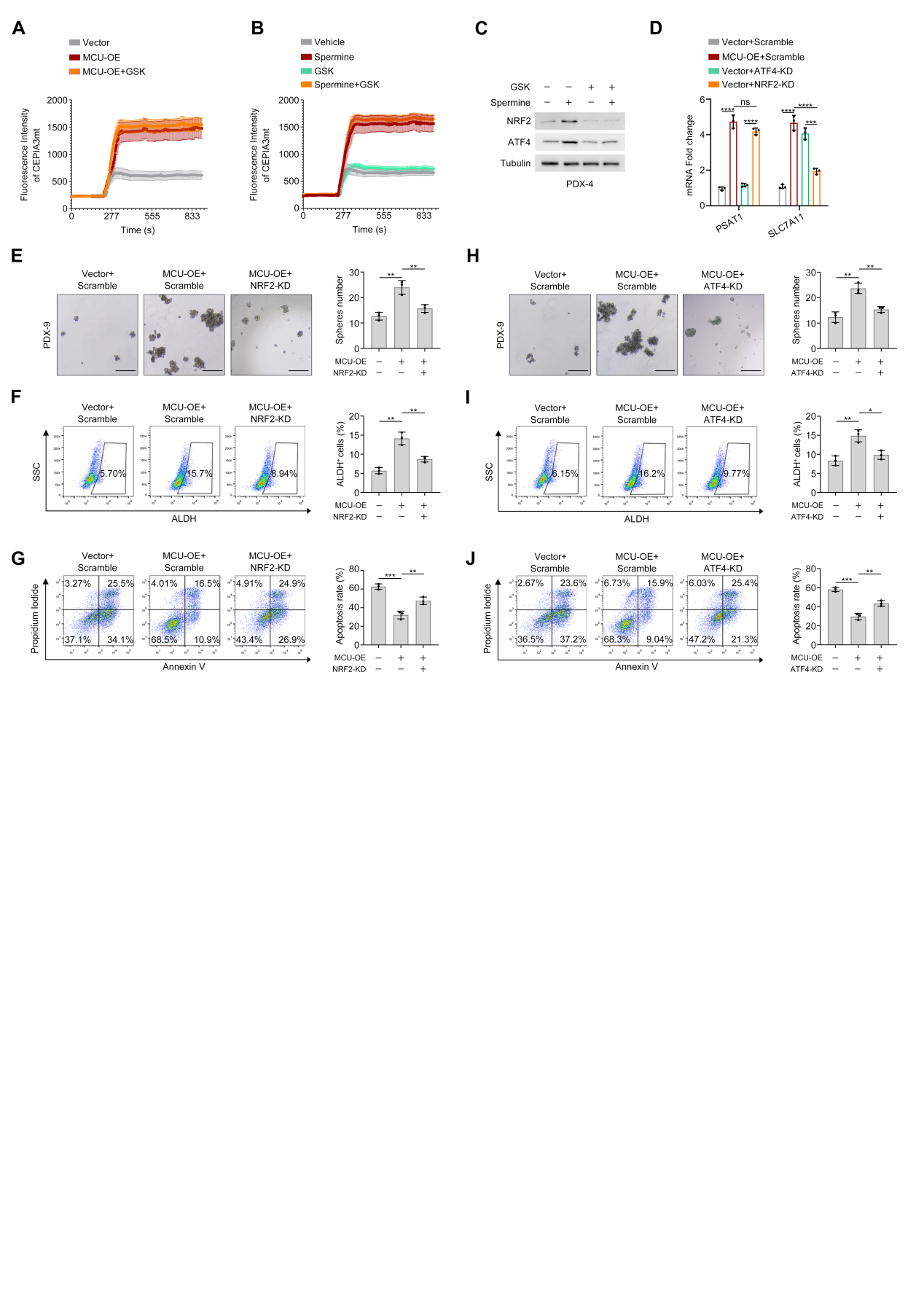
**

**Supplementary Figure S9.** **The ATF4-PSAT1 and NRF2-SLC7A11 axes synergistically contribute to MCU-mediated stemness maintenance and chemoresistance.**

(A) The effects of MCU overexpression on mitochondrial Ca^2+^ uptake after GSK2606414 treatment in PDX-4 cells. (B) The effects of MCU overexpression on mitochondrial Ca^2+^ uptake after GSK2606414 and spermine treatment on mitochondrial Ca^2+^ uptake in PDX-4 cells. (C) Western blots showing the effects of GSK2606414 and spermine on NRF2 and ATF4 expression levels in the PDX-4 cells. (D) qPCR analysis showing the effects of ATF4 knockdown and NRF2 knockdown on MCU-mediated PSAT1 and SLC7A11 expression levels in PDX-4 cells. (E-G) The effects of NRF2 knockdown on MCU-mediated stemness maintenance and chemoresistance *in vitro*. Sphere formation capacity (E), ALDH^+^ proportion (F), and apoptotic rate (G) following AG treatment of PDX-9 cells in the indicated groups are shown. Scale bar, 100 µm. (H-J) The effects of ATF4 knockdown on MCU-mediated stemness maintenance and chemoresistance *in vitro*. Sphere formation capacity (H), ALDH^+^ proportion (I), and apoptotic rate (J) following AG treatment of PDX-9 cells in the indicated groups are shown. Scale bar, 100 µm. Data in (D-J) are presented as mean ± SD of 3 biological replicates and were analyzed using two-sample, two-tailed unpaired Student’s *t*-test. ns, not significant, **P* < 0.05, ***P* < 0.01, ****P* < 0.001 and *****P* < 0.0001.


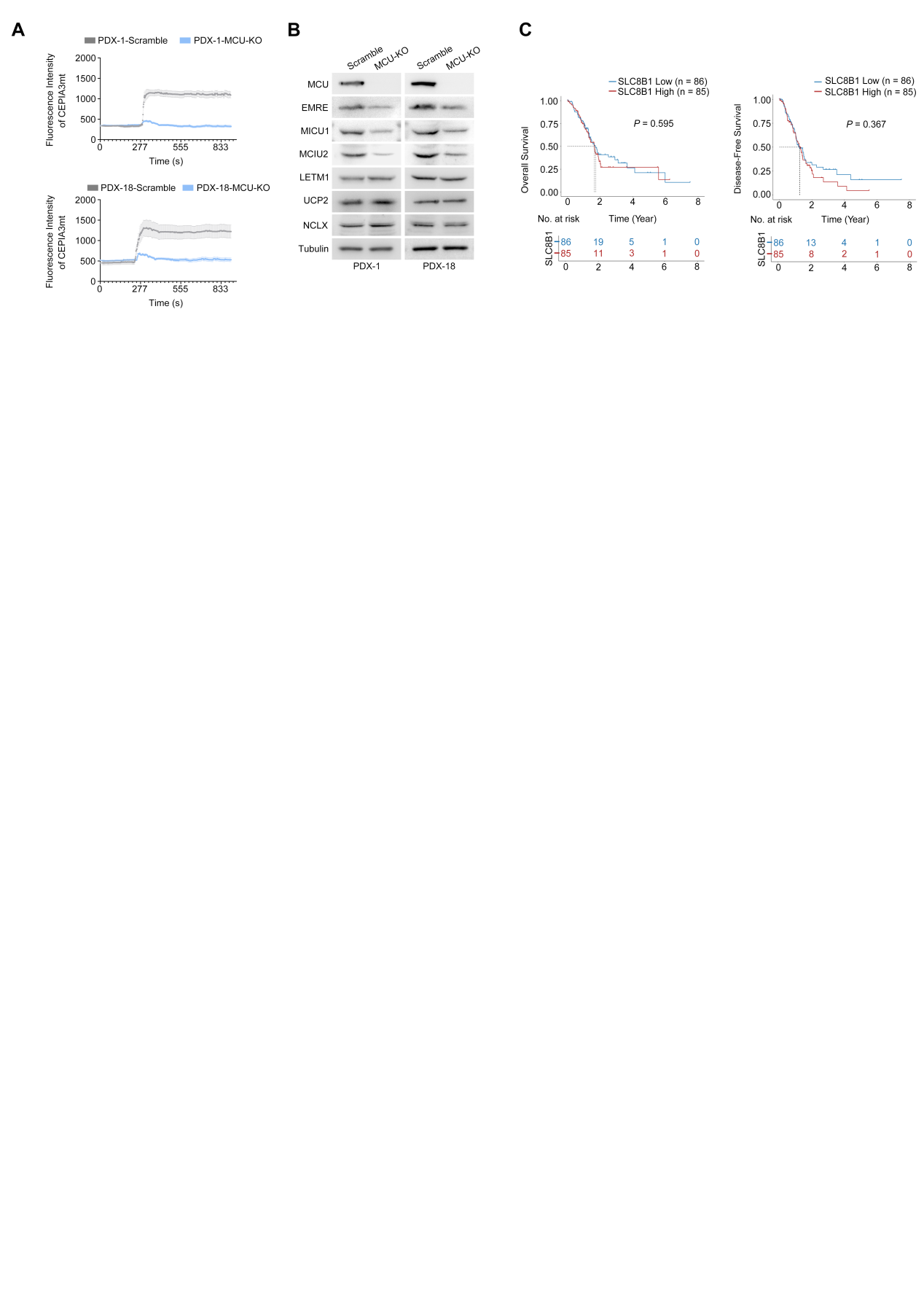


**Supplementary Figure S10. MCU loss causes irreplaceable disruption of mitochondrial Ca²⁺ uptake.**

(A) The effects of MCU knockout on mitochondrial Ca^2+^ uptake in PDX-1 and PDX-18 cells. (B) Western blots of MCU, EMRE, MICU1, MICU2, LETM1, UCP2, and NCLX expression in PDX-1 and PDX-18 cells 21 days after MCU KO. (C) Kaplan–Meier curves showing the correlation between SLC8B1 (gene encoding NCLX) mRNA expression levels and OS (left) or DFS (right) in a cohort of 171 PDAC patients from the TCGA database.

**
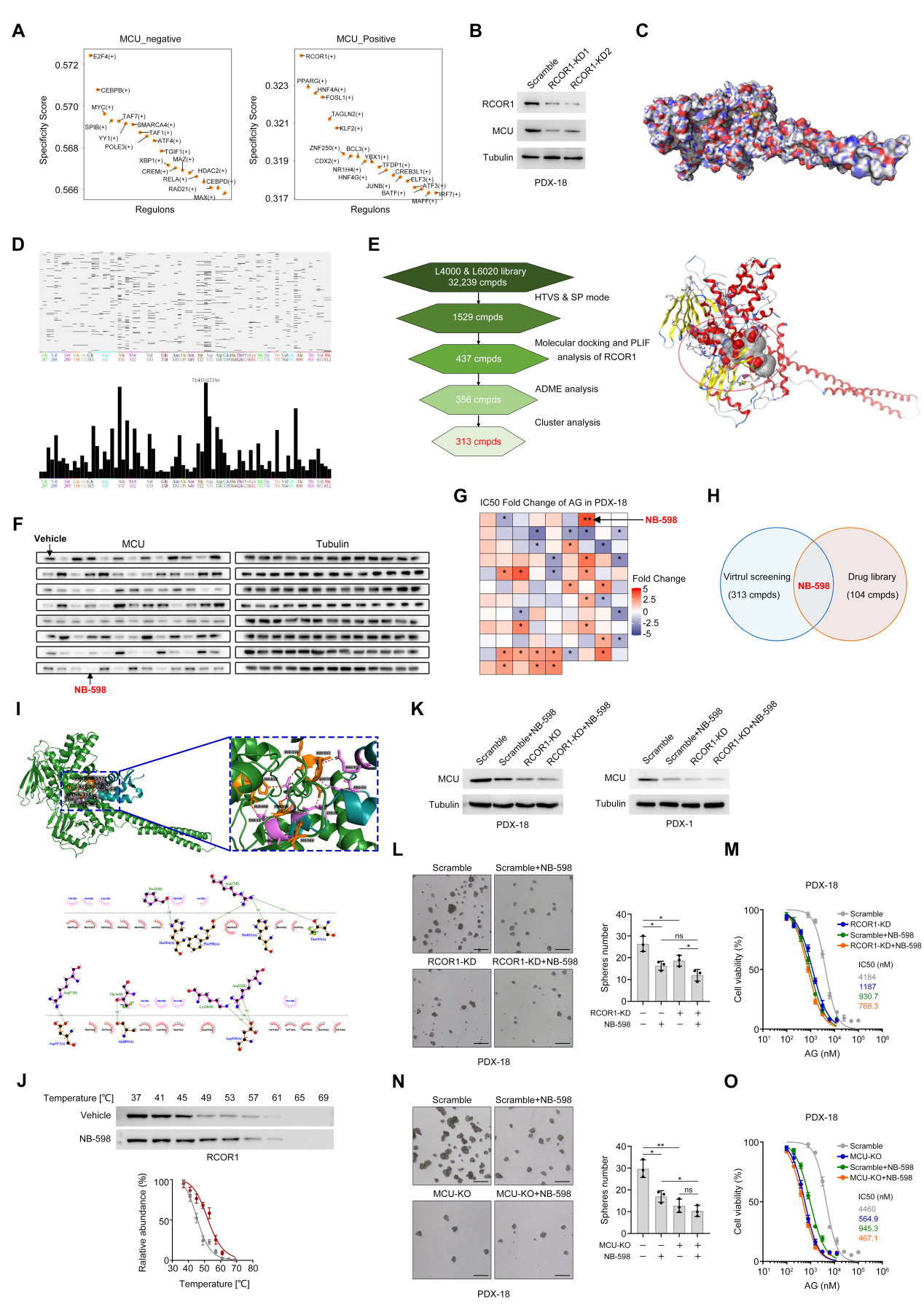
**

**Supplementary Figure S11. Identification of NB-598 as a modulator of MCU expression via AI-guided virtual screening and functional small-molecule library in PDAC cells.**

1. Scatter plots showing RCOR1 as a transcriptional regulon significantly elevated in MCU-positive cells in single-cell RNA sequencing analysis. (B) Western blots showing the effects of RCOR1 knockdown on MCU expression. (C) Schematic diagram of the surface electrostatic potential distribution of transcription factor RCOR1 (top). The crystal structure of RCOR1 and its receptor binding site are shown **(bottom).** (D) Amino acid residues involved in binding to the RCOR1 active site, with their frequency of interaction with small-molecule compounds. (E-H) Employing molecular docking-based virtual screening from the compound libraries L4000 (known active compounds) and L6020 (natural product monomer compound library) to identify lead compounds. Subsequently, assessing the rationality of small molecule compounds through structural clustering, PLIF analysis, and docking scores followed by validation through western blotting and CCK-8 assays in a screening model. (E) Hierarchical process flowchart for virtual screening of small-molecule compounds targeting RCOR1 (CoREST1). Of the 313 compounds identified through drug library screening, the lead candidate (NB-598) was validated by western blotting and CCK-8 assays (F-G). NB-598 was identified as the lead candidate (H). (I) Binding interactions of compound NB-598 with the active site of human RCOR1 (top). 2D interaction diagram illustrating specific interactions between NB-598 and RCOR1 active site residues (bottom). (J) Cellular Thermal Shift Assay (CETSA) for interaction between NB-598 and RCOR1. Thermal denaturation curve showing the abundance of bands against temperature. (K) The effects of NB-598 on MCU expression in RCOR1-KD and Scramble control PDX-18 cells. (L) The effects of NB-598 on sphere formation capacity in RCOR1-KD and Scramble control PDX-18 cells. Scale bar, 100 µm. (M) Dose response curves showing the effects of NB-598 on sensitivities to AG treatment in RCOR1-KD and Scramble control PDX cells. (N) The effects of NB-598 on sphere formation capacity in MCU-KO and Scramble control PDX-18 cells. Scale bar, 100 µm. (O) Dose response curves showing the effects of NB-598 on sensitivities to AG treatment in MCU-KO and Scramble control PDX-18 cells. Data in (G), (J-K), and (L) are presented as mean ± SD of 3 biological replicates and were analyzed using two-sample, two-tailed unpaired Student’s *t*-test. ns, not significant, **P* < 0.05, ***P* < 0.01, ****P* < 0.001 and *****P* < 0.0001.

**
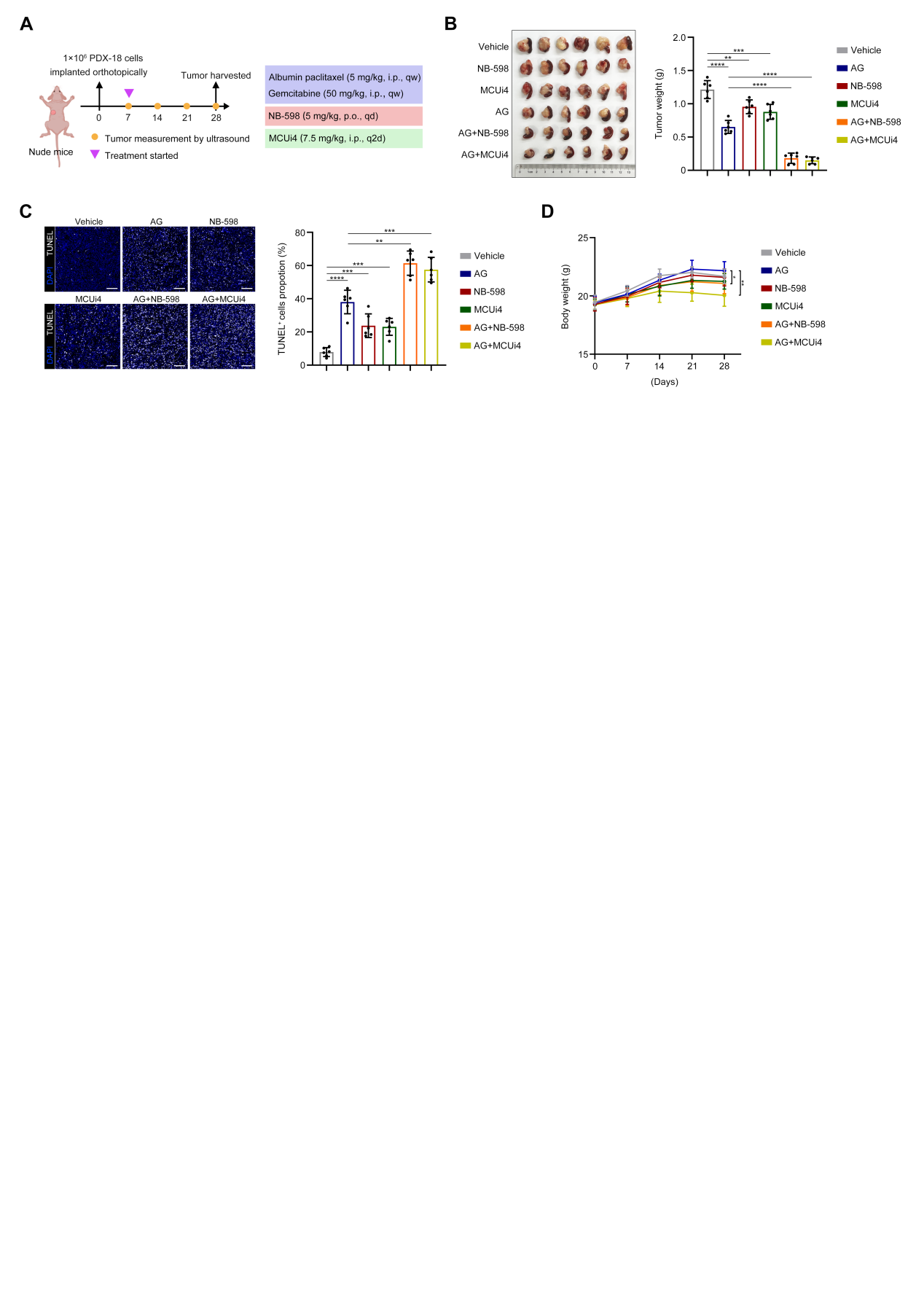
**

**Supplementary Figure S12. Inhibition of MCU effectively sensitizes PDAC to AG chemotherapy.**

1. Schematic illustration showing the experimental design. Immunocompromised BALB/c nude mice were orthotopically inoculated with 1 × 10^6^ PDX-18 cells. Mice were randomized into six groups (n = 6 per group): (i) vehicle, (ii) AG, (iii) NB-598, (iv) MCUi4, (v) AG + NB-598, and (vi) AG + MCUi4 (Albumin paclitaxel: 5 mg/kg, i.p., qw; Gemcitabine: 50 mg/kg, i.p., qw; NB-598: 5 mg/kg, p.o., qd; MCUi4: 7.5 mg/kg, i.p., q2d). (B) Representative tumor images and tumor weight of each group at Day 28 are shown. (C) Representative images of TUNEL staining in PDX tumors showing the tumor suppression effects of each group. Quantification of TUNEL^+^ cells was performed using Image J software. Scale bar: 100 µm. (D) Body weight of mice receiving vehicle, AG, NB-598, MCUi4, AG + NB-598, and AG + MCUi4. Data in (B-C) were analyzed using two sample, two-tailed, unpaired Student’s *t* test. Data in (D) were analyzed using two-way ANOVA followed by Tukey’s multiple comparison test. Data are shown as the mean value ± SD. **P* < 0.05, ***P* < 0.01, ****P* < 0.001 and *****P* < 0.0001.

**
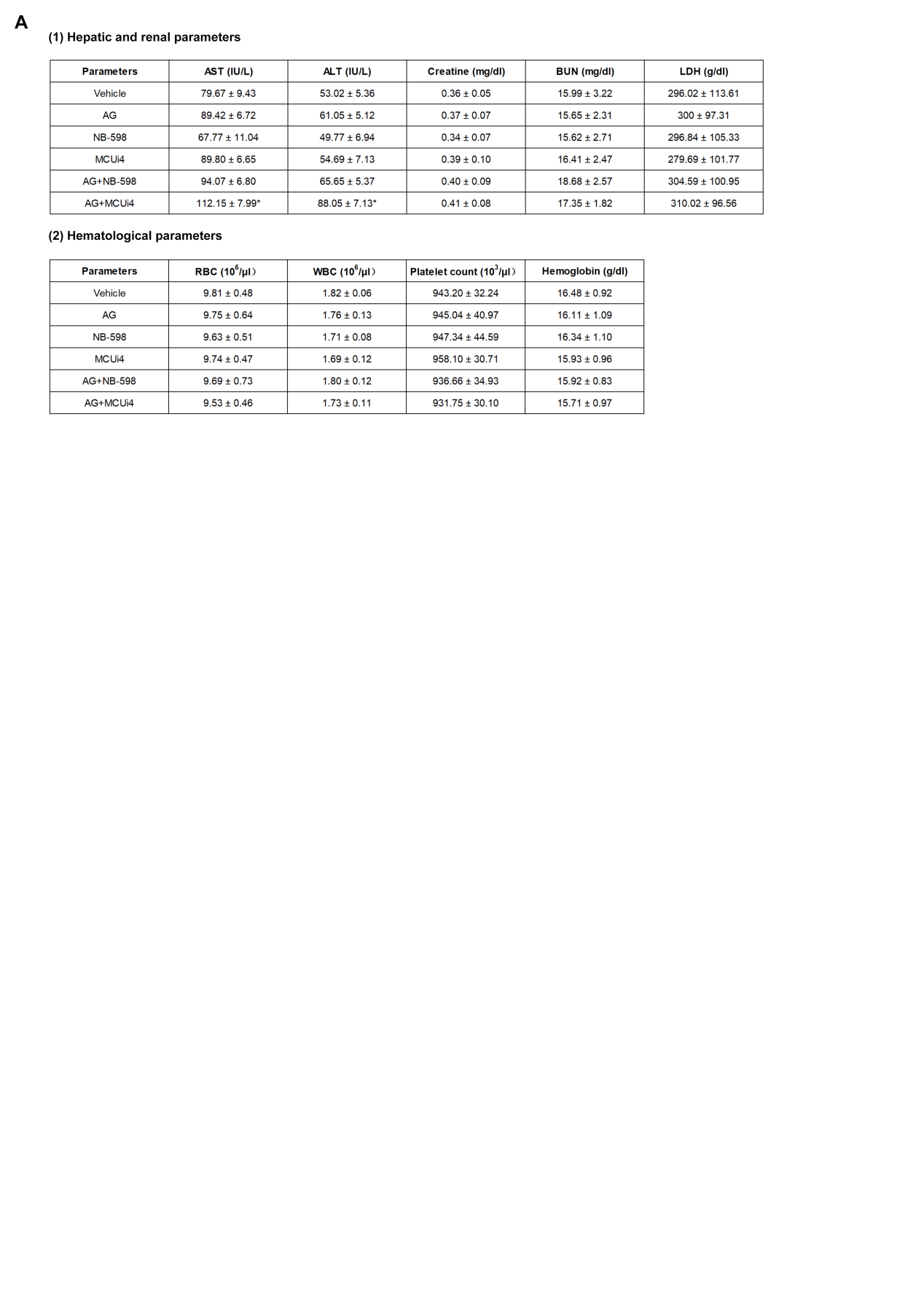
Supplementary Figure S13. Assessment of biochemical markers in tumor-bearing mice following combined administration of MCU inhibitors and AG.**

1. Evaluation of hematological parameters (1), hepatic and renal parameters (2) in mice after combined treatment (n = 6 per group). Data are presented as mean ± SD and were analyzed using two-sample, two-tailed unpaired Student’s *t*-test.

**
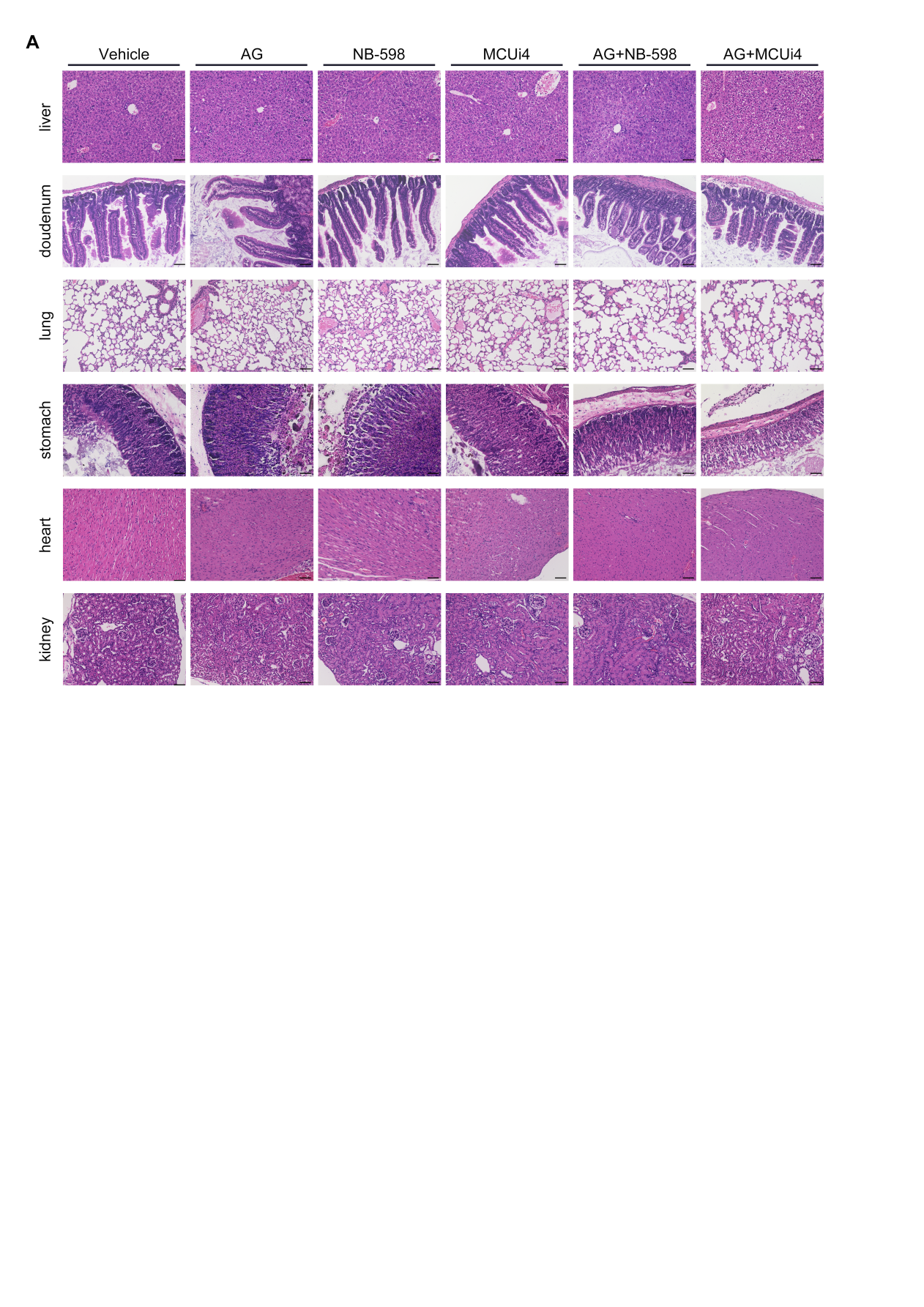
Supplementary Figure S14. Safety assessment of various organs following combined administration of MCU inhibitors and AG.**

(A) Pharmacological toxicity and safety of eight different internal organs were evaluated after combined administration in six treatment groups. Scale bar: 100 µm.


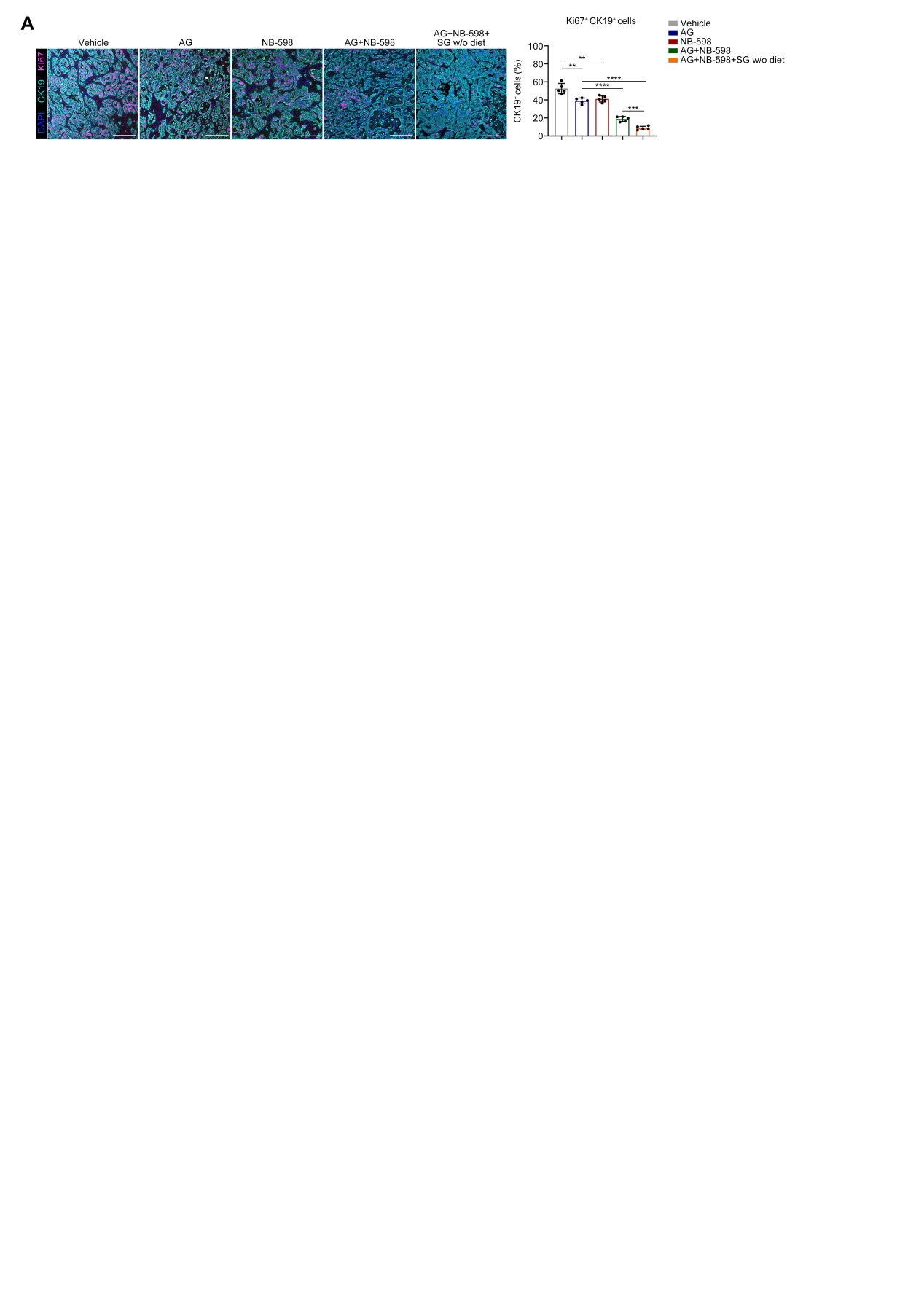


**Supplementary Figure S15. Serine-glycine dietary restriction enhances the therapeutic efficacy of combined NB-598 and AG chemotherapy.**

Representative images of Ki67 and CK19 staining in PDX tumors showing the anti-proliferative effects of each group. Quantification of Ki67^+^ CK19^+^ tumor cells was performed using Image J software. Scale bar: 100 µm. Data were analyzed using two sample, two-tailed, unpaired Student’s *t* test. Data are shown as the mean value ± SD. **P* < 0.05, ***P* < 0.01, ****P* < 0.001 and *****P* < 0.0001.
